# The T385M STAT1 gain-of-function mutation confers the most severe disease outcomes

**DOI:** 10.3389/fimmu.2025.1717692

**Published:** 2025-11-28

**Authors:** Robert Torrance, Alexander J. McKenna, Catherine King, Joe McDowell, Eleanor O’Callaghan, Jesmeen Maimaris, Adriana S. Albuquerque, Rachel Pearce, Emma C. Morris, Siobhan O. Burns

**Affiliations:** 1 Institute of Immunity and Transplantation, University College London, London, United Kingdom; 2 Department of Immunology, Royal Free London NHS Foundation Trust, London, United Kingdom; 3 Kings College London, London, United Kingdom; 4 Department of Hematology, University College London Hospitals NHS Foundation Trust, London, United Kingdom

**Keywords:** *STAT1*, gain-of-function, primary immunodeficiency, mutation, phenotype, outcome

## Abstract

**Background:**

Gain-of-function (GOF) mutations in *STAT1* cause a combined immunodeficiency characterized by chronic mucocutaneous candidiasis (CMC), recurrent infections, and autoimmunity. Mutations in the DNA-binding domain (DBD) have previously been associated with poor outcomes, but the contributions of specific variants to clinical phenotype remain unexplored.

**Methods:**

We performed a systematic literature review to identify patients with confirmed *STAT1* GOF mutations, integrating new cases with a previously reported international cohort. Clinical and genetic data were analyzed at both domain and mutation level to define genotype-phenotype correlations.

**Results:**

A total of 533 unique patients from 36 countries were identified, harboring 135 distinct mutations. As previously reported, DBD mutations were associated with increased risk of systemic infections, bronchiectasis, autoimmunity, and reduced survival. However, mutation-level stratification revealed that the T385M variant accounted for much of this effect. Compared with both other DBD mutations and mutations elsewhere in *STAT1*, T385M conferred significantly higher rates of infection, bronchiectasis, autoimmunity, and premature death (p < 0.001). Conversely, certain coiled-coil (CC) domain mutations, such as R274Q, were associated with milder disease and improved survival.

**Conclusion:**

Our findings demonstrate that the adverse prognosis previously ascribed to DBD mutations in *STAT1* GOF is predominantly driven by the T385M variant. Mutation-specific, rather than domain-level, stratification is therefore essential for accurate risk assessment and clinical management. In particular, patients predicted to have severe disease, such as those with the T385M mutation should be considered early for curative interventional therapies such as stem cell transplant or gene therapy.

## 1 Introduction

First described in 2011, germline, monoallelic gain-of-function (GOF) mutations in the Signal Transducer and Activator of Transcription 1 (STAT1) gene result in a rare, combined immunodeficiency with a severe clinical phenotype which includes chronic mucocutaneous candidiasis (CMC), often in combination with various forms of autoimmunity as well as bacterial, viral or mycobacterial infections (1–3). Over 100 mutations spread across the whole gene have been reported to date (4). The wide heterogeneity in mutation sites, clinical phenotype and disease course complicates clinical decision-making. A better understanding of genotype-phenotype correlation in patients would therefore be beneficial.

In this study, we expand on a prior meta-analysis (2) to provide an up-to-date, detailed analysis of all reported STAT1 GOF patients as identified through a systematic literature search. Our work details the largest STAT1 GOF cohort to date, with mutation-level stratification allowing for further investigation into genotype-phenotype correlations. We identify that the T385M STAT1 GOF mutation causes the most severe disease in patients and is responsible for the poor outcomes previously described in patients with mutations in the DBD (5).

## 2 Methods

### 2.1 Identification of patients for analysis

A systematic search of the MEDLINE (PubMed) literature was conducted on 21^st^ January 2025 to identify publications after 23rd June 2016 describing patients with confirmed STAT1 GOF mutations. The key search terms used were “STAT1” OR “Signal Transducer and Activator of Transcription 1” AND “gain-of-function” OR “GOF”. Data concerning a patient’s genetic mutation, age, gender, age at CMC onset, country in which they are situated, their infectious phenotype, their non-infectious clinical phenotype, and outcome was collected. To prevent the repeated inclusion of patients, the genetic, demographic, and clinical information available for each patient was manually analyzed, as well as their clinical department/team, before inclusion. When required, original case reports were retrieved to cross-check information published in case series for data quality control. Newly identified patients were combined together with the 274 patients reported by the International STAT1 GOF Study Group in 2016 (2) to create a detailed database comprising of demographic information (**Supplementary Table S1**), infectious clinical phenotypes (**Supplementary Table S2**) and non-infectious clinical phenotypes (**Supplementary Table S3**).

### 2.2 Statistical analyses

Statistical analyses were conducted using GraphPad Prism 7 (GraphPad Software Inc, USA) or STATA 18 (StatCorp. 2023. Stata Statistical Software, USA). Specifics of each statistical test used can be found in figure legends. The choice of statistical test was based upon the research question, study design, and characteristics of the data. P values were calculated using GraphPad Prism 7 or STATA and a P value of ≤ 0.05 was deemed significant. Statistical significance is displayed on figures with P values noted in the figure legends.

## 3 Results

### 3.1 Cohort description

**Figure 1:**
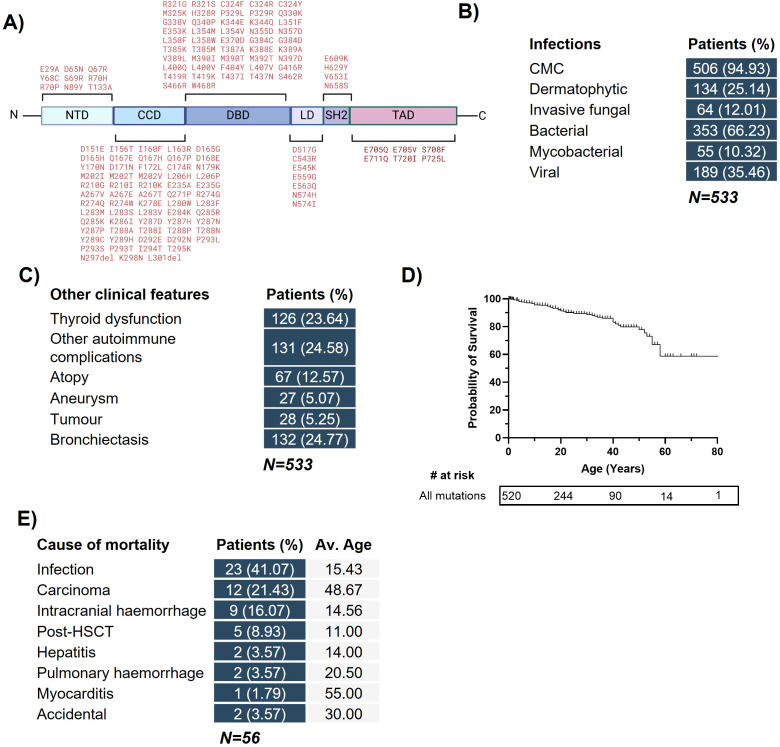
Features of patients with STAT1 gain-of-function immunodeficiency. **(A)**. Schematic of reported amino acid changes causing STAT1 GOF and the protein domain they are found in. **(B)** Breakdown of infectious clinical phenotypes in cohort. **(C)** Breakdown of non-infectious clinical phenotypes. **(D)** Kaplan-Meier survival curve for all patients in cohort. **(E)** Breakdown of the causes of mortality in patient cohort.

Our literature search identified 259 new reports of STAT1 GOF immunodeficiency, combining with the Toubiana cohort to yield 533 unique patients from 36 different countries with a confirmed molecular diagnosis of STAT1 GOF. A total of 135 distinct pathogenic mutations were identified (**Figure 1A**). When analyzed by location, the vast majority of mutations fell in the Coiled-Coil (CC; n=295) and DBD (n=191) domains, with only 47 mutations in the N-terminal, Linker, SH2 and Transactivation (TA) domains combined. *De novo* mutations were responsible for 15.2% of cases with known consanguinity in 1.7% of patients. As previously described (2), CMC, other bacterial, fungal and viral infections, bronchiectasis and autoimmune complications were common (**Figures 1B, C**). The probability of survival was substantially reduced with advancing age (**Figure 1D**), predominantly due to infection, malignancy and intracranial hemorrhage (**Figure 1E**).

### 3.2 Stratification of patients by affected domain

**Figure 2:**
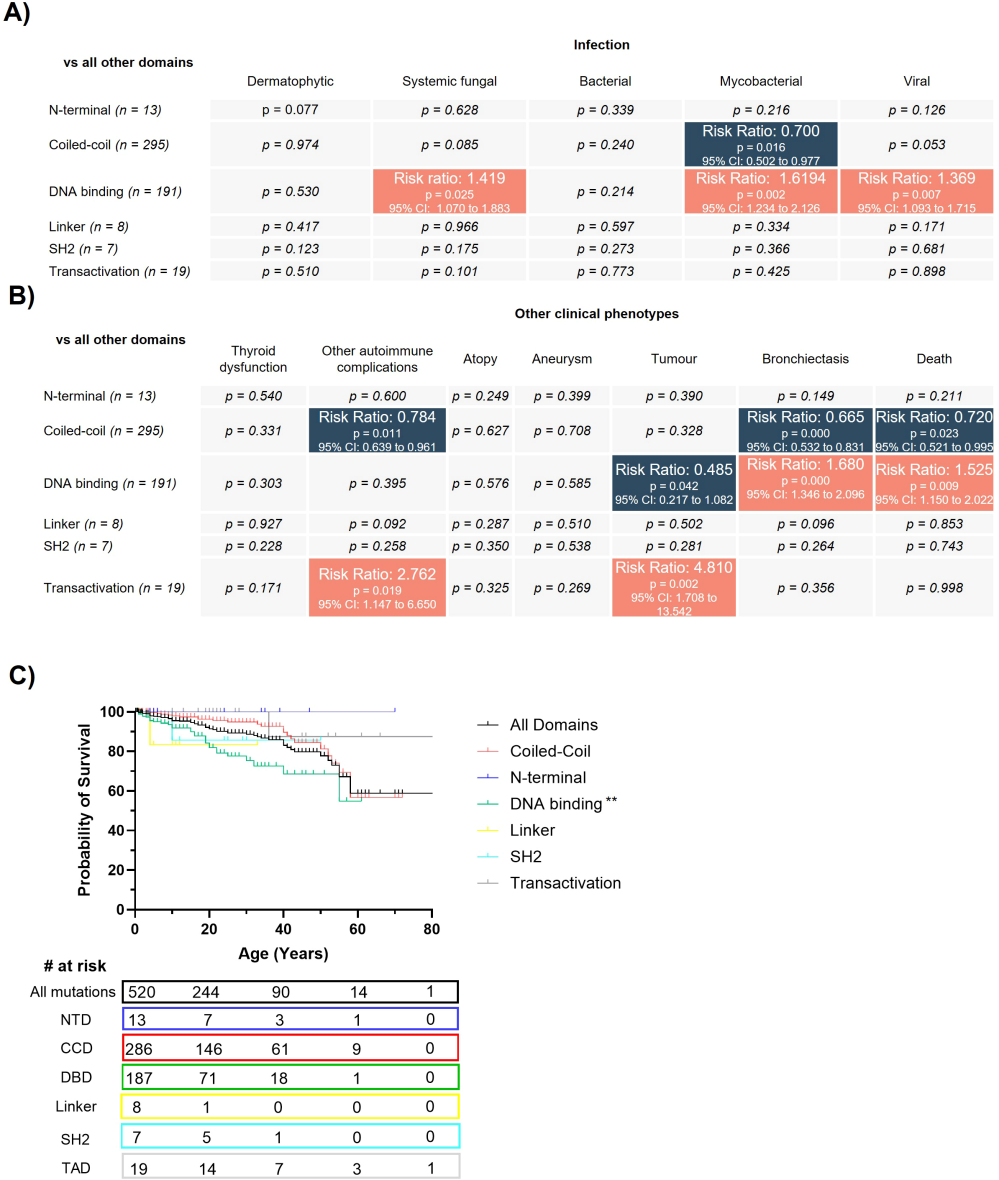
Mutations in the DNA-binding domain result in more severe disease compared to mutations in other domains. Statistical comparisons for the infectious **(A)** and non-infectious **(B)** clinical complications for patients with mutations in different STAT1 protein domains. Analysis was performed in STATA using a Pearson chi-squared test. The color scale denotes a significant decrease (blue) or increase (red) in risk (expressed as risk ratio) of that clinical complication affecting a patient with a mutation in that domain. **(C)** Kaplan-Meier survival curve for patients with STAT1 GOF mutations in different domains. Statistical comparisons between survival curves were performed using a Log-rank (Mantel-Cox) test. p<0.01(**).

Mutations in the DBD have previously been associated with more severe disease in patients at an earlier age compared with non-DBD mutations (5). In line with this, when grouping mutations by the affected domain in our cohort, mutations in the DBD were associated with significantly increased risk of infection (systemic fungal, mycobacterial and viral) and bronchiectasis compared with mutations in other domains (**Figures 2A, B**). Furthermore, although age of CMC onset and number of features per patient were broadly similar across mutations in different domains (data not shown), the probability of survival was significantly lower for patients with DBD mutations compared to patients with mutations in other domains (p=0.0039, **Figure 2C**). In keeping with this, patients with a DBD mutation were 1.5-times more at risk of death than their counterparts with mutations in other domains (**Figure 2B**). A reduced risk of tumorigenesis was also observed, presumably linked to the reduced lifespan of these patients (**Figures 2B, C**). In contrast, mutations affecting the CC domain were found to have significantly lower risk of mycobacterial infection, autoimmune complications, bronchiectasis and death (**Figures 2A, B**). Further, autoimmunity and tumors were seen more frequently in TA domain mutations, albeit with small patient numbers and 63% of cases coming from just two different families (**Figure 2B**, **Supplementary Table S1**).

Together, this data points to a more severe clinical phenotype for patients with mutations in the DBD domain in line with previous studies (5). In addition, we highlight that patients with mutations in the CC domain have a reduced risk of both infectious and non-infectious clinical phenotypes, and an overall reduced risk of death.

### 3.3 Stratification of patients by mutation

To further delineate the genotype-phenotype relationship for STAT1 GOF, we looked to examine whether any mutations were significantly contributing to the observed differences between domains. Of the 135 different mutations reported to confer STAT1 GOF, a small number of individual mutations were commonly seen, with more than 45 clinical cases for each mutation (**Supplementary Table S1**). In addition to the T385M DBD mutation (n=52), three CC domain mutations, A267V, R274Q and R274W, were frequently observed (n= 63, 54 and 46 respectively). We therefore tested whether these specific mutations predict the clinical phenotypes described above.

**Figure 3:**
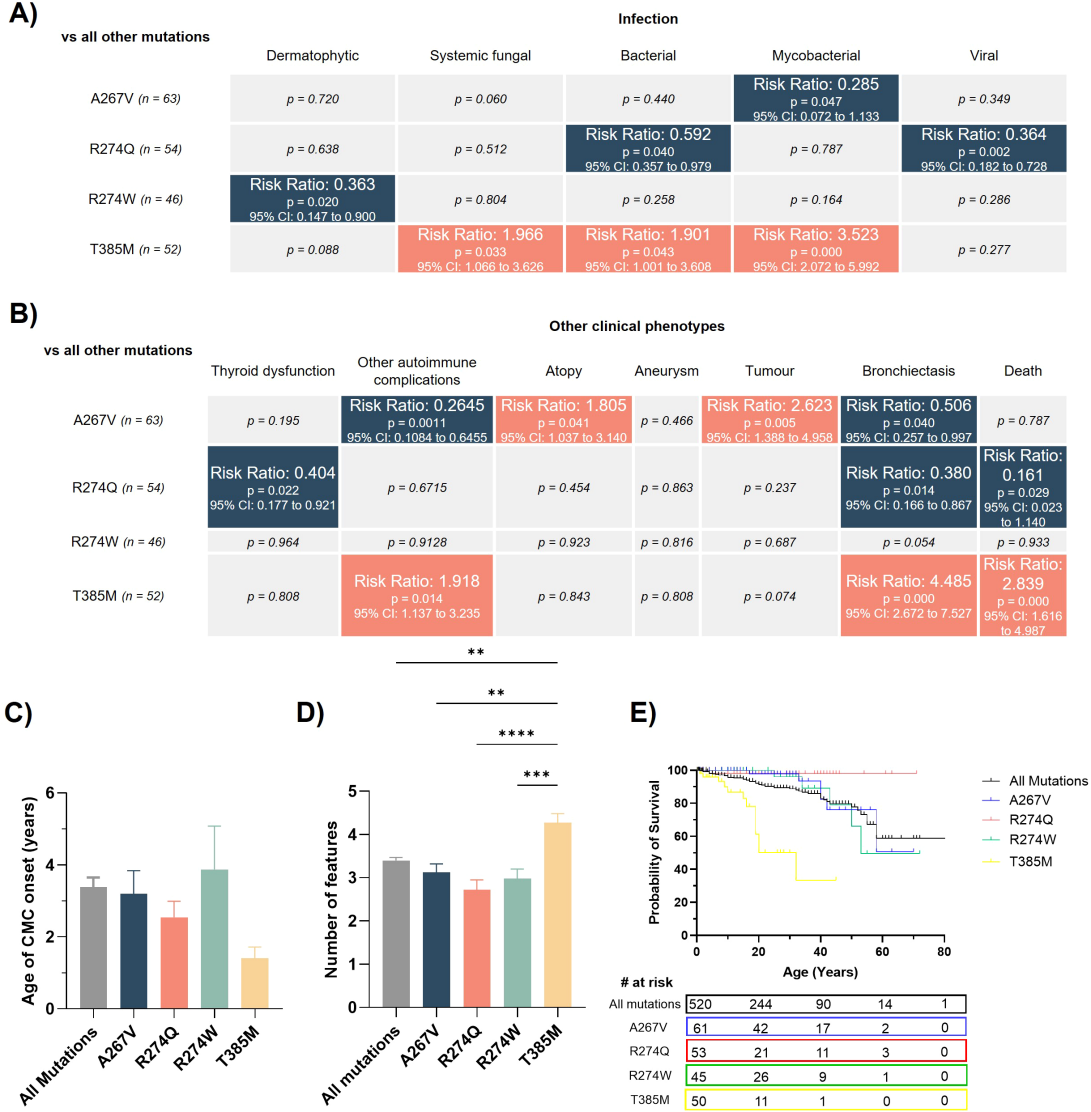
T385M is more severe and confers worse outcomes than other common STAT1 GOF mutations. Statistical comparisons for the infectious **(A)** and non-infectious **(B)** clinical complications for patients with the most common STAT1 GOF mutations (A267V, R274Q, R274W, and T385M). Analysis was performed in STATA using a Pearson chi-squared test. The color scale denotes a significant decrease (blue) or increase (red) in risk (expressed as risk ratio) of that clinical complication affecting a patient with that mutation. C) Age of CMC onset for patients with the most common STAT1 GOF mutations. **(D)** Number of clinical features for patients with the most common STAT1 GOF mutations. **(E)** Kaplan-Meier survival curve for patients with the most common STAT1 GOF mutations. Statistical comparisons between survival curves were performed using a Log-rank (Mantel-Cox) test. For **(C, D)**, statistical significance was calculated by one-way ANOVA, comparing the mean of each column with the mean of every other column. Only comparisons with p<0.05 are shown. p<0.01(**); p<0.001(***); p<0.0001 (****).

In keeping with analysis of the whole DBD cohort, patients bearing T385M mutations had significantly higher rates of systemic fungal, bacterial and mycobacterial infections, autoimmunity, bronchiectasis and death when compared with all other mutations (**Figures 3A, B**). T385M also had a trend towards earlier onset (**Figure 3C**) with significantly more clinical features (**Figure 3D**), higher rates of death (**Figure 3B**) and lower probability of survival (p<0.0001, **Figure 3E**). Of the three CC mutations only A267V had significantly higher rates of particular features, with over-representation of atopy and tumors (**Figure 3B**). Interestingly, the R274Q mutation conferred significantly reduced risk of bacterial and viral infection, thyroid dysfunction, bronchiectasis and a higher probability of survival (**Figures 3A, B, E**).

**Figure 4:**
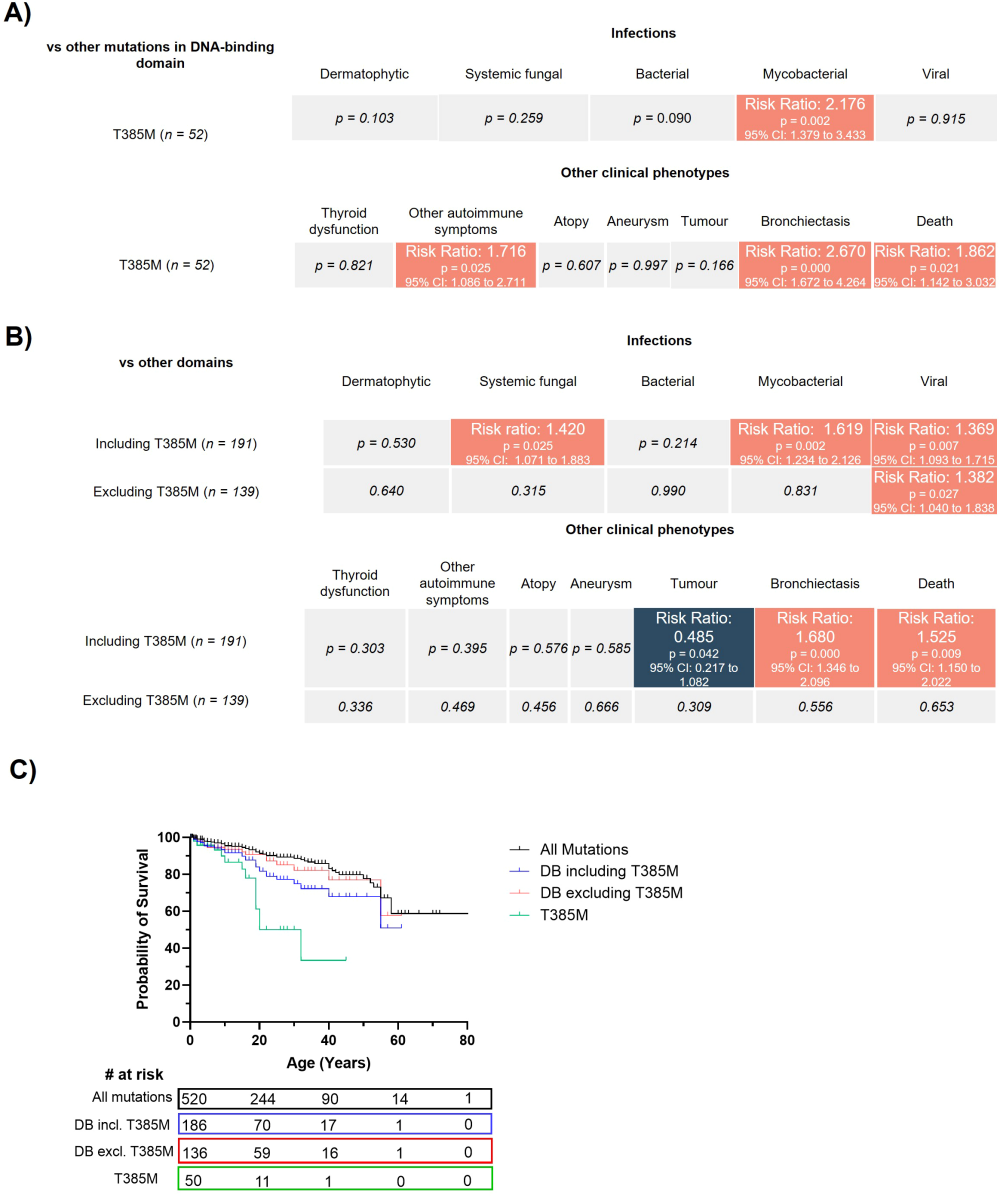
T385M is responsible for the severe clinical phenotype associated with mutations in the DBD. **(A)**. Statistical comparisons for the infectious and non-infectious clinical complications for patients with the T385M mutation compared with other mutations in the DBD. Analysis was performed in STATA using a Pearson chi-squared test. The color scale denotes a significant decrease (blue) or increase (red) in risk (expressed as risk ratio) of that clinical complication affecting a patient with T385M compared with other mutations in the DBD. **(B)** Statistical comparisons for the infectious and non-infectious clinical complications for patients with mutations in the DBD including or excluding T385M compared to mutations in other STAT1 protein domains. The color scale denotes a significant decrease (blue) or increase (red) in risk (expressed as risk ratio) of that clinical complication affecting a patient with a DBD mutation, including or excluding T385M, compared with a patient with a mutation in any other STAT1 domain. **(C)**. Kaplan-Meier survival curve for patients with mutations in the DBD, either including or excluding patients with T385M mutations. Statistical comparisons between survival curves were performed using a Log-rank (Mantel-Cox) test.

Given that patients with the T385M mutation make up over one quarter of patients with mutations in the DBD, we investigated whether the more severe phenotype associated with DBD mutations were skewed by the T385M mutation. To this end, a comparison of T385M vs other DBD mutations was conducted. Compared with other DBD mutations, T385M had significantly greater probability of having mycobacterial infections, autoimmunity, bronchiectasis and mortality (**Figure 4A**). This suggests that variability in phenotype can occur amongst mutations within the same domain. Further, we also compared the DBD mutation group including and excluding T385M (n=191 and n=139 respectively) with mutations in all other domains. Following exclusion of T385M, the DBD mutation group were not significantly different from mutations in other domains with respect to individual disease complications, apart from the incidence of viral infection (**Figure 4B**). Furthermore, T385M conferred significantly worse probability of survival compared with all other DBD mutations (p value = 0.0006, **Figure 4C**). Together, this data indicates that T385M is the predominant contributor to the severe clinical phenotype observed in patients with mutations in the DBD.

## 4 Discussion

Here, we present the largest cohort of STAT1 GOF patients to date, with detailed information regarding the demographics, clinical manifestations and outcomes of 533 patients with 135 unique GOF mutations in *STAT1*. Our updated cohort showed similar proportions of patients suffering from infectious and non-infectious clinical phenotypes compared with previous studies (2, 6) (**Figures 1B, C**), with CMC present in the vast majority of patients. Further, in line with a previous report that mutations in the DBD confer more severe disease, (5), we demonstrate that this is also the case in our cohort, with DBD mutations corresponding to increased susceptibility to fungal, bacterial and viral infection, an increased risk of bronchiectasis, and higher probability of death when compared to mutations in all other domains (**Figure 2**).

However, here, we extend this genotype-phenotype relationship beyond just the affected domain to the level of individual mutations. Specifically, we identify that the T385M variant found within the DBD confers a more severe clinical phenotype with a greater number of clinical features, significantly higher rates of severe infection, bronchiectasis, autoimmunity and premature death when compared with other common mutations (**Figure 3**). Further, we show that T385M is responsible for the severe disease that has been previously attributed to mutations within the DBD (5). When patients with the T385M mutation were removed from the DBD cohort, phenotype severity was no worse than mutations in any other domain, save for the incidence of viral infection (**Figure 4**). In contrast, certain CC mutations such as R274Q were associated with milder clinical presentations and improved survival.

While our clinical analysis indicates that T385M is associated with a markedly more severe phenotype compared with other STAT1 GOF mutations, the molecular reasons underpinning this discrepancy remain poorly understood. Recent studies have demonstrated unique, mutation-specific routes to pathogenesis for several STAT1 GOF mutations (e.g. R274W, R321S, T419R and N574I) (7, 8). However, there are currently no published mechanistic studies that characterize the mechanisms leading to the more severe clinical outcomes observed in patients with the T385M mutation.

Our results suggest that the functional impact of STAT1 GOF mutations differs, even within a given STAT1 protein domain. Clarifying the reasons for this variation will improve understanding of the disease and selection of tailored therapies in the future. Indeed, consideration of mutation-specific, rather than domain-level variation in STAT1 GOF patients will be important for improved decisions surrounding therapeutic interventions. For example, for patients expected to have milder disease such as those with the R274Q mutation, more conservative clinical management (e.g. using Janus Kinase inhibitors (JAKi) (9)) may be most appropriate. In view of recent improvements in hematopoietic stem cell transplant (HSCT) outcomes for STAT1 GOF (10), compared with historical data (11), patients with severe mutations such as T385M should be considered, not only for targeted therapy with JAKi, but also for early curative treatment options such as HSCT or gene therapies as these become available.

## Data Availability

The original contributions presented in the study are included in the article/**Supplementary Material**. Further inquiries can be directed to the corresponding author.
