# Supplementary material for: The T385M STAT1 gain-of-function mutation confers the most severe disease outcomes
Source: Front Immunol. 2025 Nov 28;16:1717692. doi: 10.3389/fimmu.2025.1717692 (PMC12698592; doi:10.3389/fimmu.2025.1717692)
Supplement: Supplementary file 1 [file Table1.docx]

**Table S1: Demographic information of each STAT1 GOF patient.**

1 indicates ‘yes’. 0 indicated ‘no’. NR indicates ‘not reported’. NP indicates ‘not present’. CMC Onset if not otherwise explicitly stated: “At birth” = 0.1; “Early Childhood” = 3; “Childhood” = 6, “Adolescence” = 13, “Adulthood” = 18;

| **Mutation** | **Domain** | **Gender** | **Age at time of study (years)** | **Age of CMC onset (years)** | **De novo** | **Familial history** | **Known consanguinity** | **Country of living** | **References** |
| --- | --- | --- | --- | --- | --- | --- | --- | --- | --- |
| E29A | N | M | 47 | NR | 1 | 0 | 0 | Czech Republic | Parackova et al., 2023  (PMID: 37358695) |
| D65N | N | F | 5 | 0.5 | 0 | 1 | 0 | Canada | Scott et al, 2022  (PMID: 35126392) |
| D65N | N | F | 39 | 18 | 0 | 1 | 0 | Canada | Scott et al, 2022  (PMID: 35126392) |
| D65N | N | F | 70 | 5 | 0 | 0 | 0 | Canada | Scott et al, 2022  (PMID: 35126392) |
| D65N | N | M | 5 | NR | 0 | 0 | 0 | China | Chen et al, 2019  (PMID: 31686315) |
| Q67R | N | F | 5 | 4 | 0 | 0 | 0 | Greece | Antoniadi et al, 2024  (PMID: 31686315) |
| Y68C | N | F | 24 | NR |  | 1 | 0 | Czech Republic | Parackova et al., 2023  (PMID: 37358695) |
| Y68C | N | F | 47 | NR | 0 | 0 | 0 | Czech Republic | Parackova et al., 2023  (PMID: 37358695) |
| S69R | N | M | 4 | 1 | 0 | 0 | 0 | Spain | Bonino et al, 2024  (PMID: 38085069) |
| R70P | N | F | 34 | 3 | 0 | 1 | 0 | UK | Carey et al, 2019  (PMID: 29702748) |
| R70H | N | F | 35 | 8 | 0 | 0 | 0 | UK | Toubiana et al, 2016  (PMID: 39413163) |
| N89Y | N | M | 6 | 0.58 | 1 | 0 | 0 | Morocco | Toubiana et al, 2016  (PMID: 39413163) |
| T133A | N | M | 6 | NR | 0 | 1 | 1 | Spain | Rudilla et al, 2019  (PMID: 31681265) |
| D151E | CC | F | 13 | 4 | 0 | 1 | 0 | Spain | Toubiana et al, 2016  (PMID: 39413163) |
| D151E | CC | M | 23 | 1 | 0 | 1 | 0 | Spain | Toubiana et al, 2016  (PMID: 39413163) |
| I156T | CC | F | 15 | 11 | 0 | 1 | 0 | USA | Toubiana et al, 2016  (PMID: 39413163) |
| I156T | CC | M | 24 | 16 | 0 | 1 | 0 | USA | Toubiana et al, 2016  (PMID: 39413163) |
| I156T | CC | F | 36 | NR | 0 | 0 | 0 | USA | Zhang et al, 2017  (PMID: 28710273) |
| I156T | CC | F | 39 | NP | 0 | 1 | 0 | USA | Toubiana et al, 2016  (PMID: 39413163) |
| I156T | CC | F | 42 | 0.5 | 0 | 1 | 0 | USA | Toubiana et al, 2016  (PMID: 39413163) |
| I156T | CC | F | 43 | NP | 0 | 1 | 0 | USA | Toubiana et al, 2016  (PMID: 39413163) |
| I156T | CC | F | 61 | NP | 0 | 1 | 0 | USA | Toubiana et al, 2016  (PMID: 39413163) |
| I160F | CC | F | 14 | 2 | 1 | 0 | 0 | France | Toubiana et al, 2016  (PMID: 39413163) |
| L163R | CC | F | 14 | 7 | 0 | 0 | 1 | Tunisia | Toubiana et al, 2016  (PMID: 39413163) |
| D165H | CC | F | 9 | NR | 1 | 0 | 0 | China | Chen et al, 2024  (PMID: 38758476) |
| D165H | CC | F | 8 | 0.5 | 0 | 0 | 0 | Germany | Toubiana et al, 2016  (PMID: 39413163) |
| D165G | CC | M | NR | NR | 0 | 0 | 0 | Netherlands | Leiding et al, 2018  (PMID: 28601685) |
| D165G | CC | M | 21 | 0.25 | 0 | 0 | 0 | Ukraine | Liu et al, 2011  (PMID: 21727188) |
| D165H | CC | M | 30 | 0.5 | 0 | 0 | 0 | USA | Zimmerman et al, 2019  (PMID: 31354696) |
| Q167H | CC | F | 2 | NR | 0 | 0 | 0 | Argentina | Bernasconi et al, 2017  (PMID: 30302727) |
| Q167H | CC | F | 9 | 1 | 0 | 0 | 0 | Argentina | Toubiana et al, 2016  (PMID: 39413163) |
| Q167E | CC | F | 51 | NR | 0 | 1 | 0 | Germany | Toubiana et al, 2016  (PMID: 39413163) |
| Q167P | CC | M | 6 | 0.25 | 0 | 1 | 0 | Italy | Toubiana et al, 2016  (PMID: 39413163) |
| Q167P | CC | F | 44 | 2 | 0 | 1 | 0 | Italy | Toubiana et al, 2016  (PMID: 39413163) |
| D168E | CC | M | 44 | 3 | 0 | 1 | 0 | Australia | Van Zelm et al 2019  (PMID: 31068927) |
| D168E | CC | F | 5 | 2 | 1 | 0 | 1 | Morocco | Toubiana et al, 2016  (PMID: 39413163) |
| Y170N | CC | M | 9 | 0.92 | 1 | 0 | 0 | Switzerland | Toubiana et al, 2016  (PMID: 39413163) |
| D171N | CC | M | 5 | 0.67 | 0 | 0 | 0 | France | Baghad et al, 2019  (PMID: 31677808) |
| D171N | CC | M | 26 | 0.66 | 0 | 1 | 0 | Germany | Toubiana et al, 2016  (PMID: 39413163) |
| F172L | CC | M | 10 | 3 | 0 | 0 | 0 | Germany | Kobbe et al 2016  (PMID: 27063510) |
| F172L | CC | F | 25 | 16 | 0 | 0 | 0 | USA | Sampaio et al 2013  (PMID: 23541320) |
| C174R | CC | F | 15 | 0.1 | 0 | 1 | 0 | Germany | Toubiana et al, 2016  (PMID: 39413163) |
| C174R | CC | F | 18 | 2 | 0 | 1 | 0 | Germany | Toubiana et al, 2016  (PMID: 39413163) |
| C174R | CC | M | 19 | 1 | 0 | 1 | 0 | Germany | Toubiana et al, 2016  (PMID: 39413163) |
| C174R | CC | F | 40 | 2 | 0 | 1 | 0 | Germany | Toubiana et al, 2016  (PMID: 39413163) |
| C174R | CC | F | 46 | 2 | 0 | 1 | 0 | Germany | Toubiana et al, 2016  (PMID: 39413163) |
| C174R | CC | F | 49 | 0.75 | 0 | 1 | 0 | Germany | Toubiana et al, 2016  (PMID: 39413163) |
| C174R | CC | M | 58 | 1 | 0 | 1 | 0 | Germany | Toubiana et al, 2016  (PMID: 39413163) |
| C174R | CC | F | 32 | NR | 0 | 1 | 0 | Italy | Solimando et al, 2023  (PMID: 36826612) |
| N179K | CC | F | 9 | 6 | 0 | 0 | 0 | Czech Republic | Toubiana et al, 2016  (PMID: 39413163) |
| M202V | CC | M | 24 | 0.17 | 0 | 0 | 0 | Brazil | Toubiana et al, 2016  (PMID: 39413163) |
| M202T | CC | F | 19 | 0.5 | 0 | 0 | 0 | China | Wang et al, 2016  (PMID: 27808400) |
| M202I | CC | M | 8 | 0.08 | 0 | 1 | 0 | France | Toubiana et al, 2016  (PMID: 39413163) |
| M202I | CC | M | 11 | 1 | 0 | 1 | 0 | France | Toubiana et al, 2016  (PMID: 39413163) |
| M202V | CC | M | 16 | 0.1 | 0 | 1 | 0 | France | Toubiana et al, 2016  (PMID: 39413163) |
| M202V | CC | M | 30 | 1.5 | 0 | 1 | 0 | France | Toubiana et al, 2016  (PMID: 39413163) |
| M202V | CC | M | 35 | 5 | 0 | 1 | 0 | France | Olivier et al, 2022  (PMID: 35696308) |
| M202V | CC | F | 37 | 10 | 0 | 1 | 0 | France | Toubiana et al, 2016  (PMID: 39413163) |
| M202I | CC | F | 46 | 0.5 | 0 | 1 | 0 | France | Toubiana et al, 2016  (PMID: 39413163) |
| M202V | CC | M | 55 | 4 | 0 | 1 | 0 | France | Toubiana et al, 2016  (PMID: 39413163) |
| M202T | CC | F | 6 | 0.08 | 0 | 0 | 0 | Japan | Toubiana et al, 2016  (PMID: 39413163) |
| M202V | CC | M | 37 | 1 | 0 | 0 | 0 | Japan | Toubiana et al, 2016  (PMID: 39413163) |
| M202I | CC | F | 35 | 5.5 | 1 | 0 | 0 | Spain | Olbrich et al, 2023  (PMID: 36881346) |
| M202V | CC | F | 10 | NR | 0 | 0 | 0 | USA | Forbes et al, 2018  (PMID: 30092289) |
| M202I | CC | F | 15 | NR | 0 | 0 | 0 | USA | Vargas-Hernandez et al, 2018  (PMID: 29111217) |
| L206P | CC | M | 12 | 0.17 | 1 | 0 | 0 | Czech Republic | Bloomfield et al 2018  (PMID: 29934865) |
| L206H | CC | M | 6 | 1.33 | 0 | 0 | 0 | India | Toubiana et al, 2016  (PMID: 39413163) |
| R210G | CC | F | 6 | 0.1 | 1 | 0 | 0 | Netherlands | Toubiana et al, 2016  (PMID: 39413163) |
| R210K | CC | M | 34 | 2 | 1 | 0 | 0 | UK | Toubiana et al, 2016  (PMID: 39413163) |
| R210K | CC | F | 6 | NR | 0 | 0 | 0 | USA | Kunvarjee et al, 2023  (PMID: 37367708) |
| R210I | CC | M | 12 | 0.5 | 0 | 0 | 0 | USA | Toubiana et al, 2016  (PMID: 39413163) |
| E235A | CC | F | 34 | NR | 0 | 0 | 0 | USA | Zhang et al, 2017  (PMID: 28710273) |
| E235G | CC | F | 34 | 0.02 | 0 | 0 | 0 | USA | Zimmerman et al, 2019  (PMID: 31354696) |
| E235A | CC | F | 60 | NR | 0 | 0 | 0 | USA | Zhang et al, 2017  (PMID: 28710273) |
| E235G | CC | F | 62 | 0.5 | 0 | 0 | 0 | USA | Zimmerman et al, 2019  (PMID: 31354696) |
| A267E | CC | M | 5 | NP | 0 | 0 | 0 | Argentina | Bernasconi et al, 2017  (PMID: 30302727) |
| A267V | CC | M | 6 | 0.25 | 0 | 0 | 0 | Australia | Hosking et al, 2020  (PMID: 32146551) |
| A267V | CC | F | NR | NR | 0 | 0 | 0 | Australia | Hosking et al, 2020  (PMID: 32146551) |
| A267V | CC | M | 8 | 1 | 1 | 0 | 0 | Belgium | Toubiana et al, 2016  (PMID: 39413163) |
| A267T | CC | M | 11 | NR | 0 | 1 | 0 | Canada | Garkaby et al, 2021  (DOI: [doi.org/10.14785/lymphosign-2021-0019](https://doi.org/10.14785/lymphosign-2021-0019)) |
| A267T | CC | M | 11 | NR | 0 | 1 | 0 | Canada | Garkaby et al, 2021  (DOI: [doi.org/10.14785/lymphosign-2021-0019](https://doi.org/10.14785/lymphosign-2021-0019)) |
| A267T | CC | F | 14 | 4 | 0 | 1 | 0 | Canada | Garkaby et al, 2021  (DOI: [doi.org/10.14785/lymphosign-2021-0019](https://doi.org/10.14785/lymphosign-2021-0019)) |
| A267T | CC | M | 48 | NR | 0 | 1 |  | Canada | Garkaby et al, 2021  (DOI: [doi.org/10.14785/lymphosign-2021-0019](https://doi.org/10.14785/lymphosign-2021-0019)) |
| A267V | CC | F | 4 | 0.58 | 0 | 1 | 0 | Chile | Toubiana et al, 2016  (PMID: 39413163) |
| A267V | CC | F | 27 | 2 | 0 | 1 | 0 | Chile | Toubiana et al, 2016  (PMID: 39413163) |
| A267V | CC | F | 13 | 1 | 0 | 1 | 0 | Czech Republic | Toubiana et al, 2016  (PMID: 39413163) |
| A267V | CC | F | 22 | NR | 1 | 0 | 0 | Czech Republic | Parackova et al., 2023  (PMID: 37358695) |
| A267V | CC | F | 40 | 1 | 0 | 1 | 0 | Czech Republic | Toubiana et al, 2016  (PMID: 39413163) |
| A267V | CC | F | 48 | NR | 1 | 0 | 0 | Czech Republic | Parackova et al., 2023  (PMID: 37358695) |
| A267V | CC | M | 70 | NR | 0 | 1 | 0 | Czech Republic | Toubiana et al, 2016  (PMID: 39413163) |
| A267V | CC | M | NR | NR | 0 | 1 | 0 | Czech Republic | Toubiana et al, 2016  (PMID: 39413163) |
| A267V | CC | M | 1 | 0.33 | 0 | 1 | 0 | France | Toubiana et al, 2016  (PMID: 39413163) |
| A267V | CC | F | 16 | 1.5 | 1 | 0 | 0 | France | Toubiana et al, 2016  (PMID: 39413163) |
| A267V | CC | M | 34 | 2 | 0 | 1 | 0 | France | Toubiana et al, 2016  (PMID: 39413163) |
| A267V | CC | M | 46 | 20 | 0 | 0 | 0 | France | Toubiana et al, 2016  (PMID: 39413163) |
| A267V | CC | F | 27 | 20 | 0 | 0 | 0 | Germany | Dadak et al, 2017  (PMID: 28161409) |
| A267V | CC | F | 27 | 1 | 0 | 0 | 0 | Germany | Toubiana et al, 2016  (PMID: 39413163) |
| A267V | CC | F | 1 | 1 | 0 | 1 | 0 | Iran | Toubiana et al, 2016  (PMID: 39413163) |
| A267V | CC | M | 31 | 1 | 0 | 1 | 0 | Iran | Toubiana et al, 2016  (PMID: 39413163) |
| A267V | CC | M | 26 | 3 | 0 | 1 | 0 | Israel | Breuer et al 2017  (PMID: 28427548) |
| A267V | CC | M | 27 | 1 | 0 | 1 | 0 | Israel | Toubiana et al, 2016  (PMID: 39413163) |
| A267V | CC | M | 29 | 1 | 0 | 1 | 0 | Israel | Toubiana et al, 2016  (PMID: 39413163) |
| A267V | CC | M | 46 | 1 | 0 | 1 | 0 | Israel | Toubiana et al, 2016  (PMID: 39413163) |
| A267V | CC | F | 14 | 0.5 | 0 | 0 | 0 | Italy | Dotta et al, 2016  (PMID: 26732859) |
| A267V | CC | M | 2 | 0.17 | 0 | 1 | 0 | Japan | Toubiana et al, 2016  (PMID: 39413163) |
| A267V | CC | M | 13 | 1.5 | 0 | 1 | 0 | Japan | Toubiana et al, 2016  (PMID: 39413163) |
| A267V | CC | F | 43 | 1 | 0 | 1 | 0 | Japan | Toubiana et al, 2016  (PMID: 39413163) |
| A267V | CC | F | 48 | 1 | 0 | 1 | 0 | Japan | Toubiana et al, 2016  (PMID: 39413163) |
| A267V | CC | F | 24 | NR | 1 | 0 | 0 | Korea | Huh et al, 2018  (PMID: 30187709) |
| A267V | CC | M | 6 | 0.33 | 1 | 0 | 0 | Morocco | Toubiana et al, 2016  (PMID: 39413163) |
| A267V | CC | M | 19 | 5 | 0 | 1 | 0 | Netherlands | Toubiana et al, 2016  (PMID: 39413163) |
| A267V | CC | F | 33 | 0.75 | 0 | 1 | 0 | Netherlands | Toubiana et al, 2016  (PMID: 39413163) |
| A267V | CC | F | 53 | 1 | 0 | 1 | 0 | Netherlands | Toubiana et al, 2016  (PMID: 39413163) |
| A267V | CC | M | 23 | 1 | 0 | 1 | 0 | Norway | Toubiana et al, 2016  (PMID: 39413163) |
| A267V | CC | F | 31 | 1 | 0 | 1 | 0 | Norway | Toubiana et al, 2016  (PMID: 39413163) |
| A267V | CC | M | 48 | 3 | 0 | 1 | 0 | Norway | Toubiana et al, 2016  (PMID: 39413163) |
| A267V | CC | F | 36 | NR | 0 | 1 | 0 | Sweden | Borgstrom et al, 2022  (PMID: 36050429) |
| A267V | CC | F | 53 | NR | 0 | 0 | 0 | Sweden | Borgstrom et al, 2022  (PMID: 36050429) |
| A267V | CC | F | 10 | 0.5 | 0 | 1 | 0 | UK | Toubiana et al, 2016  (PMID: 39413163) |
| A267V | CC | M | 11 | 0.1 | 0 | 1 | 0 | UK | Toubiana et al, 2016  (PMID: 39413163) |
| A267V | CC | F | 12 | 3 | 0 | 1 | 0 | UK | Koo et al, 2017  (PMID: 28815025) |
| A267V | CC | F | 21 | 6 | 0 | 1 | 0 | UK | Koo et al, 2017  (PMID: 28815025) |
| A267V | CC | F | 31 | 5 | 0 | 1 | 0 | UK | Toubiana et al, 2016  (PMID: 39413163) |
| A267V | CC | M | 31 | 13 | 0 | 1 | 0 | UK | Toubiana et al, 2016  (PMID: 39413163) |
| A267V | CC | M | 32 | 3 | 0 | 1 | 0 | UK | Koo et al, 2017  (PMID: 28815025) |
| A267V | CC | F | 34 | 6 | 0 | 1 | 0 | UK | Koo et al, 2017  (PMID: 28815025) |
| A267V | CC | M | 36 | NR | 0 | 1 | 0 | UK | Toubiana et al, 2016  (PMID: 39413163) |
| A267V | CC | M | 40 | 3 | 0 | 1 | 0 | UK | Koo et al, 2017  (PMID: 28815025) |
| A267V | CC | M | 40 | 3 | 0 | 1 | 0 | UK | Toubiana et al, 2016  (PMID: 39413163) |
| A267V | CC | F | 41 | NR | 0 | 1 | 0 | UK | Toubiana et al, 2016  (PMID: 39413163) |
| A267V | CC | M | 42 | 4 | 0 | 1 | 0 | UK | Toubiana et al, 2016  (PMID: 39413163) |
| A267V | CC | M | 56 | 2 | 0 | 0 | 0 | UK | Toubiana et al, 2016  (PMID: 39413163) |
| A267V | CC | F | 58 | 3 | 0 | 1 | 0 | UK | Koo et al, 2017  (PMID: 28815025) |
| A267V | CC | F | 63 | 15 | 0 | 1 | 0 | UK | Toubiana et al, 2016  (PMID: 39413163) |
| A267V | CC | M | 0.75 | 0 | 1 | 0 | 0 | USA | Kaviany et al, 2022  (PMID: 35840326) |
| A267V | CC | F | 17 | NP | 0 | 0 | 0 | USA | Toubiana et al, 2016  (PMID: 39413163) |
| A267V | CC | M | 18 | 6 | 0 | 1 | 0 | USA | Rosenberg et al, 2022  (PMID: 35590143) |
| A267V | CC | M | 19 | 0.5 | 0 | 0 | 0 | USA | Zimmerman et al, 2019  (PMID: 31354696) |
| A267V | CC | M | 21 | 0.5 | 0 | 1 | 0 | USA | Toubiana et al, 2016  (PMID: 39413163) |
| A267V | CC | M | 23 | 0.75 | 0 | 1 | 0 | USA | Toubiana et al, 2016  (PMID: 39413163) |
| A267V | CC | F | 24 | 1.92 | 0 | 1 | 0 | USA | Toubiana et al, 2016  (PMID: 39413163) |
| A267V | CC | F | 25 | 13 | 1 | 0 | 0 | USA | Toubiana et al, 2016  (PMID: 39413163) |
| A267V | CC | F | 30 | 2 | 0 | 0 | 0 | USA | Toubiana et al, 2016  (PMID: 39413163) |
| Q271P | CC | F | 41 | 1 | 0 | 0 | 0 | Germany | Liu et al, 2011  (PMID: 21727188) |
| Q271P | CC | F | 22 | 0.75 | 1 | 0 | 0 | Iran | Ostadi et al, 2021  (PMID: 32526033) |
| Q271P | CC | F | 31 | 13 | 0 | 0 | 0 | Iran | Salemi et al, 2024  (PMID: 39023634) |
| Q271P | CC | F | 7 | 3 | 0 | 0 | 1 | Morocco | Toubiana et al, 2016  (PMID: 39413163) |
| Q271P | CC | F | 12 | 0.5 | 1 | 0 | 1 | Morocco | Baghad et al 2021  (PMID: 33404972) |
| R274Q | CC | M | 6 | NR | 0 | 0 | 0 | Argentina | Bernasconi et al, 2017  (PMID: 30302727) |
| R274W | CC | M | 13 | NR | 0 | 0 | 0 | Argentina | Bernasconi et al, 2017  (PMID: 30302727) |
| R274W | CC | F | 15 | 0.1 | 0 | 1 | 0 | Argentina | Toubiana et al, 2016  (PMID: 39413163) |
| R274W | CC | M | 18 | 0.1 | 0 | 1 | 0 | Argentina | Toubiana et al, 2016  (PMID: 39413163) |
| R274W | CC | F | 38 | 0.1 | 0 | 1 | 0 | Argentina | Toubiana et al, 2016  (PMID: 39413163) |
| R274Q | CC | F | 45 | 1 | 1 | 0 | 0 | Brazil | Oliveira Rekowski et al, 2024  (PMID: 39499953) |
| R274Q | CC | M | 4 | 0 | 1 | 0 | 0 | Brazil | Lyra et al, 2022  (PMID: 36102410) |
| R274Q | CC | F | 18 | 2 | 0 | 1 | 0 | Brazil | Oliveira Rekowski et al, 2024  (PMID: 39499953) |
| R274Q | CC | F | 25 | 3 | 0 | 0 | 0 | Chile | Toubiana et al, 2016  (PMID: 39413163) |
| R274W | CC | F | 2 | 1 |  | 1 | 0 | China | Liu et al, 2023  (PMID: 36683786) |
| R274Q | CC | M | 5 | NR | 0 | 0 | 0 | China | Chen et al, 2019  (PMID: 31686315) |
| R274Q | CC | F | 20 | 2 | 0 | 0 | 0 | China | Wang et al, 2016  (PMID: 27808400) |
| R274Q | CC | M | 29 | 4 | 0 | 0 | 0 | China | Wang et al, 2016  (PMID: 27808400) |
| R274Q | CC | F | 3 | 0.1 | 0 | 1 | 0 | Dubai | Toubiana et al, 2016  (PMID: 39413163) |
| R274Q | CC | M | 5 | 0.1 | 0 | 1 | 0 | Dubai | Toubiana et al, 2016  (PMID: 39413163) |
| R274Q | CC | F | 32 | 1 | 0 | 1 | 0 | Dubai | Toubiana et al, 2016  (PMID: 39413163) |
| R274Q | CC | F | 13 | 0.17 | 0 | 1 | 0 | France | Toubiana et al, 2016  (PMID: 39413163) |
| R274W | CC | F | 13 | 0.1 | 0 | 1 | 0 | France | Toubiana et al, 2016  (PMID: 39413163) |
| R274Q | CC | F | 16 | 0.08 | 0 | 1 | 0 | France | Toubiana et al, 2016  (PMID: 39413163) |
| R274W | CC | M | 18 | 0.1 | 0 | 1 | 0 | France | Toubiana et al, 2016  (PMID: 39413163) |
| R274Q | CC | M | 33 | 0.1 | 0 | 1 | 0 | France | Toubiana et al, 2016  (PMID: 39413163) |
| R274W | CC | F | 36 | 0.1 | 0 | 1 | 0 | France | Toubiana et al, 2016  (PMID: 39413163) |
| R274W | CC | F | 36 | 1.5 | 1 | 0 | 0 | France | Toubiana et al, 2016  (PMID: 39413163) |
| R274Q | CC | F | 38 | 1 | 0 | 1 | 0 | France | Toubiana et al, 2016  (PMID: 39413163) |
| R274W | CC | M | 50 | 10 | 0 | 1 | 0 | France | Toubiana et al, 2016  (PMID: 39413163) |
| R274Q | CC | M | 54 | 0.1 | 0 | 1 | 0 | France | Toubiana et al, 2016  (PMID: 39413163) |
| R274Q | CC | F | 63 | 7 | 0 | 1 | 0 | France | Toubiana et al, 2016  (PMID: 39413163) |
| R274Q | CC | F | 61 | 1 | 0 | 0 | 0 | France | Toubiana et al, 2016  (PMID: 39413163) |
| R274Q | CC | F | 12 | 10 | 0 | 1 | 0 | Germany | Toubiana et al, 2016  (PMID: 39413163) |
| R274Q | CC | M | 14 | 0.25 | 0 | 1 | 0 | Germany | Toubiana et al, 2016  (PMID: 39413163) |
| R274Q | CC | F | 14 | 10 | 0 | 1 | 0 | Germany | Toubiana et al, 2016  (PMID: 39413163) |
| R274Q | CC | M | 17 | 4 | 0 | 0 | 0 | Germany | Haake et al, 2020  (PMID: 32000109) |
| R274W | CC | M | 23 | 3 | 0 | 1 | 0 | Germany | Toubiana et al, 2016  (PMID: 39413163) |
| R274W | CC | F | 25 | 3 | 0 | 0 | 0 | Germany | Toubiana et al, 2016  (PMID: 39413163) |
| R274W | CC | M | 27 | 3 | 0 | 1 | 0 | Germany | Toubiana et al, 2016  (PMID: 39413163) |
| R274Q | CC | M | 39 | 1 | 0 | 0 | 0 | Germany | Toubiana et al, 2016  (PMID: 39413163) |
| R274Q | CC | F | 39 | 3 | 0 | 1 | 0 | Germany | Toubiana et al, 2016  (PMID: 39413163) |
| R274W | CC | F | 53 | 0.33 | 1 | 0 | 0 | Germany | Toubiana et al, 2016  (PMID: 39413163) |
| R274W | CC | F | 55 | NR | 0 | 1 | 0 | Germany | Toubiana et al, 2016  (PMID: 39413163) |
| R274Q | CC | M | NR | NR | 0 | 1 | 0 | Germany | Toubiana et al, 2016  (PMID: 39413163) |
| R274Q | CC | M | 10 | 10 | 0 | 1 | 0 | Greece | Toubiana et al, 2016  (PMID: 39413163) |
| R274Q | CC | F | 40 | 3 | 0 | 1 | 0 | Greece | Toubiana et al, 2016  (PMID: 39413163) |
| R274Q | CC | F | 18 | 1.08 | 0 | 0 | 0 | Hungary | Toubiana et al, 2016  (PMID: 39413163) |
| R274W | CC | F | 18 | 1 | 0 | 1 | 0 | Hungary | Toubiana et al, 2016  (PMID: 39413163) |
| R274W | CC | F | 50 | 3 | 0 | 1 | 0 | Hungary | Toubiana et al, 2016  (PMID: 39413163) |
| R274Q | CC | F | 4 | 0.66 | 0 | 1 | 0 | Iran | Ostadi et al, 2021  (PMID: 32526033) |
| R274Q | CC | M | 18 | 1 | 0 | 1 | 0 | Iran | Ostadi et al, 2021  (PMID: 32526033) |
| R274Q | CC | F | 5 | 3 | 0 | 0 | 0 | Israel | Molho-Pessach et al, 2020  (PMID: 31637766) |
| R274Q | CC | F | 14 | 0.1 | 0 | 0 | 0 | Israel | Molho-Pessach et al, 2020  (PMID: 31637766) |
| R274Q | CC | M | 44 | 6 | 0 | 0 | 0 | Israel | Molho-Pessach et al, 2020  (PMID: 31637766) |
| R274Q | CC | F | 12 | NP | 0 | 0 | 0 | Israel | Molho-Pessach et al, 2020  (PMID: 31637766) |
| R274Q | CC | F | 6 | 1 | 0 | 1 | 0 | Japan | Toubiana et al, 2016  (PMID: 39413163) |
| R274W | CC | M | 30 | 27 | 0 | 0 | 0 | Japan | Andou et al, 2023  (PMID: 37779067) |
| R274W | CC | M | 30 | 30 | 0 | 1 | 0 | Japan | Nemoto et al, 2020  (PMID: 33133069) |
| R274Q | CC | F | 35 | 1 | 0 | 1 | 0 | Japan | Toubiana et al, 2016  (PMID: 39413163) |
| R274W | CC | M | 12 | 3 | 0 | 0 | 0 | Morocco | Toubiana et al, 2016  (PMID: 39413163) |
| R274Q | CC | M | 2 | 0.25 | 0 | 1 | 0 | Netherlands | Toubiana et al, 2016  (PMID: 39413163) |
| R274Q | CC | M | 2 | 0.25 | 0 | 1 | 0 | Netherlands | Toubiana et al, 2016  (PMID: 39413163) |
| R274Q | CC | F | 6 | 0.1 | 0 | 1 | 0 | Netherlands | Toubiana et al, 2016  (PMID: 39413163) |
| R274Q | CC | M | 10 | 5 | 0 | 1 | 0 | Netherlands | Toubiana et al, 2016  (PMID: 39413163) |
| R274Q | CC | M | 14 | 5 | 0 | 1 | 0 | Netherlands | Toubiana et al, 2016  (PMID: 39413163) |
| R274Q | CC | M | 41 | 3 | 0 | 1 | 0 | Netherlands | Toubiana et al, 2016  (PMID: 39413163) |
| R274Q | CC | M | 42 | 12 | 0 | 1 | 0 | Netherlands | Toubiana et al, 2016  (PMID: 39413163) |
| R274Q | CC | M | 43 | NR | 0 | 0 | 0 | Netherlands | Zimmmerman et al, 2017  (PMID: 29226168) |
| R274Q | CC | M | 46 | 0.75 | 0 | 1 | 0 | Netherlands | Toubiana et al, 2016  (PMID: 39413163) |
| R274Q | CC | M | 71 | 6 | 0 | 1 | 0 | Netherlands | Toubiana et al, 2016  (PMID: 39413163) |
| R274W | CC | M | 72 | NR | 0 | 0 | 0 | Netherlands | Zimmmerman et al, 2017  (PMID: 29226168) |
| R274W | CC | F | 25 | 1 | 0 | 1 | 0 | Norway | Toubiana et al, 2016  (PMID: 39413163) |
| R274W | CC | M | 32 | 1 | 0 | 1 | 0 | Norway | Toubiana et al, 2016  (PMID: 39413163) |
| R274W | CC | F | NR | NR | 0 | 0 | 0 | Norway | Toubiana et al, 2016  (PMID: 39413163) |
| R274W | CC | M | 1 | 0.83 | 1 | 0 | 0 | Turkey | Toubiana et al, 2016  (PMID: 39413163) |
| R274G | CC | M | 8 | 3 | 0 | 0 | 1 | Turkey | Toubiana et al, 2016  (PMID: 39413163) |
| R274W | CC | M | 10 | 1 | 0 | 1 | 0 | Turkey | Toubiana et al, 2016  (PMID: 39413163) |
| R274W | CC | M | 29 | 1.5 | 0 | 0 | 1 | Turkey | Toubiana et al, 2016  (PMID: 39413163) |
| R274W | CC | M | 34 | NR | 0 | 1 | 0 | Turkey | Toubiana et al, 2016  (PMID: 39413163) |
| R274Q | CC | F | 1.75 | 0 | 0 | 0 | 0 | UK | Hadžić et al, 2024  (PMID: 38231631) |
| R274W | CC | M | 10 | 2 | 0 | 1 | 0 | UK | Toubiana et al, 2016  (PMID: 39413163) |
| R274Q | CC | M | 12 | NR | 0 | 1 | 0 | UK | Rae et al 2017  (PMID: 28983403) |
| R274Q | CC | M | 14 | NR | 0 | 1 | 0 | UK | Rae et al 2017  (PMID: 28983403) |
| R274W | CC | M | 15 | 0.1 | 0 | 1 | 0 | UK | Toubiana et al, 2016  (PMID: 39413163) |
| R274W | CC | F | 15 | NR | 0 | 1 | 0 | UK | Toubiana et al, 2016  (PMID: 39413163) |
| R274W | CC | M | 16 | 12 | 0 | 1 | 0 | UK | Toubiana et al, 2016  (PMID: 39413163) |
| R274W | CC | F | 27 | 1 | 0 | 1 | 0 | UK | Toubiana et al, 2016  (PMID: 39413163) |
| R274W | CC | M | 27 | 1 | 0 | 1 | 0 | UK | Toubiana et al, 2016  (PMID: 39413163) |
| R274W | CC | M | 36 | 2 | 0 | 1 | 0 | UK | Toubiana et al, 2016  (PMID: 39413163) |
| R274W | CC | M | 43 | 0.1 | 0 | 1 | 0 | UK | Toubiana et al, 2016  (PMID: 39413163) |
| R274W | CC | M | 45 | 13 | 0 | 1 | 0 | UK | Toubiana et al, 2016  (PMID: 39413163) |
| R274W | CC | F | 49 | 5 | 0 | 1 | 0 | UK | Toubiana et al, 2016  (PMID: 39413163) |
| R274W | CC | F | 55 | NR | 0 | 1 | 0 | UK | Toubiana et al, 2016  (PMID: 39413163) |
| R274W | CC | F | 3 | NR | 0 | 0 | 0 | USA | Largent et al, 2023  (PMID: 37406138) |
| R274W | CC | M | 3 | NR | 0 | 0 | 0 | USA | Vargas-Hernandez et al, 2018  (PMID: 29111217) |
| R274Q | CC | M | 4 | 3 | 0 | 1 | 0 | USA | Zimmerman et al, 2019  (PMID: 31354696) |
| R274W | CC | F | 8 | 3 | 0 | 1 | 0 | USA | Toubiana et al, 2016  (PMID: 39413163) |
| R274W | CC | M | 10 | NR | 0 | 0 | 0 | USA | Largent et al, 2023  (PMID: 37406138) |
| R274W | CC | F | 10 | NR | 0 | 0 | 0 | USA | Vargas-Hernandez et al, 2018  (PMID: 29111217) |
| R274Q | CC | M | 13 | 0.5 | 0 | 1 | 0 | USA | Toubiana et al, 2016  (PMID: 39413163) |
| R274Q | CC | F | 14 | 2.5 | 0 | 0 | 0 | USA | Toubiana et al, 2016  (PMID: 39413163) |
| R274Q | CC | F | 30 | 3 | 0 | 1 | 0 | USA | Zimmerman et al, 2019  (PMID: 31354696) |
| R274W | CC | F | 31 | NR | 0 | 0 | 0 | USA | Vargas-Hernandez et al, 2018  (PMID: 29111217) |
| R274W | CC | F | 32 | 0.75 | 0 | 1 | 0 | USA | Toubiana et al, 2016  (PMID: 39413163) |
| R274Q | CC | M | 14 | 0.5 | 0 | 0 | 0 |  | Kaleviste et al 2019  (PMID: 30801692) |
| K278E | CC | F | 20 | 1 | 1 | 0 | 0 | Japan | Yamazaki et al, 2014  (PMID: 25288569) |
| L280W | CC | F | 3 | 0.58 | 0 | 0 | 0 | UK | Papadopoulou et al 2020  (PMID: 30715505) |
| L283F | CC | M | 4 | NR | 0 | 0 | 0 | China | Chen et al, 2019  (PMID: 31686315) |
| L283V | CC | M | 12 | NR | 1 | 0 | 0 | China | Chen et al, 2024  (PMID: 38758476) |
| L283S | CC | F | 7 | 3 | 0 | 0 | 0 | Ireland | Smyth et al 2018  (PMID: 29804236) |
| L283M | CC | M | 11 | 1 | 0 | 0 | 0 | Italy | Dotta et al, 2016  (PMID: 26732859) |
| L283M | CC | F | 45 | 1 | 0 | 0 | 0 | Italy | Dotta et al, 2016  (PMID: 26732859) |
| E284K | CC | F | 27 | 3 |  | 1 | 0 | UK | Albuquerque et al, 2023  (PMID: 36722341) |
| E284K | CC | M | 55 | 16 |  | 1 | 0 | UK | Albuquerque et al, 2023  (PMID: 36722341) |
| E284K | CC | M | 55 | 40 | 0 | 0 | 0 | UK | Carey et al, 2019  (PMID: 29702748) |
| Q285R | CC | F | 8 | 6 | 1 | 0 | 0 | China | Xie et al, 2022  (PMID: 36105803) |
| Q285R | CC | F | 10 | 0.17 | 0 | 0 | 0 | Hungary | Toubiana et al, 2016  (PMID: 39413163) |
| Q285K | CC | M | 5 | 0.17 | 0 | 0 | 0 | USA | Largent et al, 2023  (PMID: 37406138) |
| Q285K | CC | M | 5 | NR | 0 | 0 | 0 | USA | Vargas-Hernandez et al, 2018  (PMID: 29111217) |
| K286I | CC | M | 7 | 5 | 0 | 1 | 0 | France | Toubiana et al, 2016  (PMID: 39413163) |
| K286I | CC | M | 26 | 5 | 0 | 1 | 0 | France | Toubiana et al, 2016  (PMID: 39413163) |
| K286I | CC | F | 52 | 3 | 0 | 1 | 0 | France | Toubiana et al, 2016  (PMID: 39413163) |
| Y287D | CC | M | 5 | NR | 0 | 0 | 0 | Argentina | Bernasconi et al, 2017  (PMID: 30302727) |
| Y287N | CC | M | 20 | 0.25 | 1 | 0 | 0 | China | Chen et al, 2021  (PMID: 34421897) |
| Y287H | CC | M | 15 | 1 | 0 | 0 | 0 | France | Toubiana et al, 2016  (PMID: 39413163) |
| Y287D | CC | M | 37 | 0.25 | 0 | 0 | 0 | UK | Toubiana et al, 2016  (PMID: 39413163) |
| T288I | CC | M | 54 | 10 | 0 | 0 | 0 | Canada | Toubiana et al, 2016  (PMID: 39413163) |
| T288A | CC | M | 6 | NR | 0 | 0 | 0 | China | Chen et al, 2019  (PMID: 31686315) |
| T288N | CC | F | 52 | NR | 1 | 0 | 0 | Czech Republic | Parackova et al., 2023  (PMID: 37358695) |
| T288P | CC | M | 3 | 0.25 | 1 | 0 | 0 | Egypt | Toubiana et al, 2016  (PMID: 39413163) |
| T288A | CC | F | NR | 0.5 | 0 | 0 | 0 | Israel | Tirosh et al 2019  (PMID: 31362757) |
| T288A | CC | F | 28 | 0.75 | 1 | 0 | 0 | Mexico | Toubiana et al, 2016  (PMID: 39413163) |
| T288A | CC | F | 9 | 4 | 0 | 0 | 0 | Portugal | Reis et al, 2021  (PMID: 34176198) |
| T288A | CC | F | 10 | 0.17 | 0 | 1 | 0 | Switzerland | Toubiana et al, 2016  (PMID: 39413163) |
| T288A | CC | F | 46 | 18 | 0 | 1 | 0 | Switzerland | Toubiana et al, 2016  (PMID: 39413163) |
| Y289H | CC | F | 18 | 2 | 1 | 0 | 0 | Belgium | Toubiana et al, 2016  (PMID: 39413163) |
| Y289C | CC | M | 12 | 10 | 1 | 0 | 0 | China | Lu et al, 2022  (PMID: 36225936) |
| Y289C | CC | M | 8 | 1 | 1 | 0 | 0 | China | Cao et al, 2022  (PMID: 36304533) |
| Y289C | CC | F | 14 | 11 | 0 | 1 | 0 | France | Toubiana et al, 2016  (PMID: 39413163) |
| Y289C | CC | F | 20 | 0.1 | 0 | 0 | 0 | France | Toubiana et al, 2016  (PMID: 39413163) |
| Y289C | CC | M | 36 | 11 | 1 | 0 | 0 | UK | Toubiana et al, 2016  (PMID: 39413163) |
| Y289C | CC | M | 2 | 2 | 0 | 0 | 0 | USA | Largent et al, 2023  (PMID: 37406138) |
| Y289C | CC | M | 4 | 0.21 | 0 | 0 | 0 | USA | Largent et al, 2023  (PMID: 37406138) |
| Y289C | CC | M | 5 | NR | 0 | 0 | 0 | USA | Vargas-Hernandez et al, 2018  (PMID: 29111217) |
| Y289C | CC | M | 8 | 0 | 0 | 0 | 0 | USA | Kaviany et al, 2022  (PMID: 35840326) |
| Y289C | CC | M | 8 | NP | 0 | 0 | 0 | USA | Vargas-Hernandez et al, 2018  (PMID: 29111217) |
| Y289C | CC | F | 30 | NR | 0 | 0 | 0 | USA | Vargas-Hernandez et al, 2018  (PMID: 29111217) |
| D292N | CC | F | 21 | 10 | 1 | 0 | 0 | Germany | Toubiana et al, 2016  (PMID: 39413163) |
| D292E | CC | M | 10 | 1 | 0 | 0 | 0 | Japan | Toubiana et al, 2016  (PMID: 39413163) |
| P293L | CC | M | 9 | NR | 0 | 0 | 0 | Germany | Toubiana et al, 2016  (PMID: 39413163) |
| P293L | CC | F | 3.5 | 1.42 | 1 | 0 | 0 | Peru | Toubiana et al, 2016  (PMID: 39413163) |
| P293S | CC | F | NR | NR | 0 | 1 | 0 | Slovakia | Toubiana et al, 2016  (PMID: 39413163) |
| P293S | CC | M | NR | NR | 0 | 1 | 0 | Slovakia | Toubiana et al, 2016  (PMID: 39413163) |
| P293S | CC | F | 32 | 15 |  | 0 | 0 | UK | Albuquerque et al, 2023  (PMID: 36722341) |
| P293T | CC | M | 39 | 0.06 | 0 | 0 | 0 | USA | Toubiana et al, 2016  (PMID: 39413163) |
| I294T | CC | F | 17 | NR | 0 | 0 | 0 | Canada | Sharfe et al, 2014  (PMID: 24239102) |
| I294T | CC | F | 13 | 1 | 0 | 0 | 0 | Japan | Toubiana et al, 2016  (PMID: 39413163) |
| I294T | CC | M | 27 | NR | 1 |  | 0 | Sweden | Borgstrom et al, 2022  (PMID: 36050429) |
| T295K | CC | F | 40 | 7 | 0 | 0 | 0 | France | Martinot et al, 2021  (PMID: 34987506) |
| N297del | CC | M | 4 | 0.5 | 0 | 0 | 0 | Mexico | Tello et al, 2024  (PMID: 39436495) |
| K298N | CC | M | 12 | 3 | 0 | 0 | 0 | Spain | Martinez-Martinez et al, 2015  (PMID: 26514428) |
| K298N | CC | M | 15 | 3 | 0 | 0 | 0 | Spain | Martinez-Martinez et al, 2015  (PMID: 26514428) |
| K298N | CC | M | 43 | 3 | 0 | 0 | 0 | Spain | Martinez-Martinez et al, 2015  (PMID: 26514428) |
| L301del | CC | F | 8 | 0.25 | 1 | 0 | 0 | Belgium | Toubiana et al, 2016  (PMID: 39413163) |
| R321S | DB | M | 48 | 1.5 | 0 | 0 | 0 | Belgium | Toubiana et al, 2016  (PMID: 39413163) |
| R321S | DB | M | 55 | 29 | 0 | 0 | 0 | Belgium | Staels et al, 2023  (PMID: 37020552) |
| R321G | DB | F | 26 | 0.17 | 0 | 0 | 0 | France | Toubiana et al, 2016  (PMID: 39413163) |
| R321G | DB | M | 7 | 0.25 | 1 | 0 | 0 | Italy | Toubiana et al, 2016  (PMID: 39413163) |
| R321G | DB | F | 26 | 0.5 | 0 | 0 | 0 | Japan | Toubiana et al, 2016  (PMID: 39413163) |
| R321S | DB | F | 16 | NR | 0 | 1 | 0 | USA | Toubiana et al, 2016  (PMID: 39413163) |
| R321S | DB | F | 18 | 5 | 0 | 1 | 0 | USA | Toubiana et al, 2016  (PMID: 39413163) |
| R321S | DB | F | 18 | 6 | 0 | 1 | 0 | USA | Zimmerman et al, 2019  (PMID: 31354696) |
| R321S | DB | F | 20 | NP | 0 | 0 | 0 | USA | Zimmmerman et al, 2017  (PMID: 29226168) |
| R321S | DB | F | 21 | 6 | 0 | 1 | 0 | USA | Zimmerman et al, 2019  (PMID: 31354696) |
| R321S | DB | M | 22 | 8 | 0 | 1 | 0 | USA | Toubiana et al, 2016  (PMID: 39413163) |
| R321S | DB | M | 25 | 0.5 | 0 | 1 | 0 | USA | Zimmerman et al, 2019  (PMID: 31354696) |
| R321S | DB | F | 40 | 10 | 0 | 1 | 0 | USA | Toubiana et al, 2016  (PMID: 39413163) |
| C324R | DB | M | 8 | NP | 0 | 0 | 0 | Canada | Sharfe et al, 2014  (PMID: 24239102) |
| C324R | DB | M | 7 | 0.67 | 0 | 1 | 0 | France | Toubiana et al, 2016  (PMID: 39413163) |
| C324R | DB | M | 13 | 5 | 0 | 1 | 0 | France | Toubiana et al, 2016  (PMID: 39413163) |
| C324R | DB | F | 14 | 0.67 | 0 | 1 | 0 | France | Toubiana et al, 2016  (PMID: 39413163) |
| C324R | DB | F | 43 | 0.1 | 0 | 1 | 0 | France | Toubiana et al, 2016  (PMID: 39413163) |
| C324R | DB | M | 27 | 3 | 0 | 1 | 0 | Germany | Toubiana et al, 2016  (PMID: 39413163) |
| C324R | DB | M | 61 | 0.75 | 0 | 1 | 0 | Germany | Toubiana et al, 2016  (PMID: 39413163) |
| C324R | DB | F | 25 | 25 | 0 | 0 | 0 | Japan | Maeshima et al 2019  (PMID: 30442829) |
| C324F | DB | M | 3 | 0.33 | 0 | 0 | 0 | Turkey | Baris et al 2016  (PMID: 27379765) |
| C324Y | DB | F | 11 | NR | 0 | 0 | 0 | USA | Forbes et al, 2018  (PMID: 30092289) |
| M325K | DB | M | 15 | NR | 0 | 0 | 0 | China | Chen et al, 2019  (PMID: 31686315) |
| H328R | DB | M | 7 | NR | 0 | 0 | 0 | USA | Vargas-Hernandez et al, 2018  (PMID: 29111217) |
| H328R | DB | M | 8 | 0.42 | 0 | 0 | 0 | USA | Largent et al, 2023  (PMID: 37406138) |
| H328R | DB | M | 10 | NP | 0 | 0 | 0 | USA | Leiding et al, 2018  (PMID: 28601685) |
| P329L | DB | M | 16 | NR | 0 | 0 | 0 | Argentina | Bernasconi et al, 2017  (PMID: 30302727) |
| P329L | DB | F | 3.5 | 1 | 0 | 0 | 0 | China | Wang et al, 2016  (PMID: 27808400) |
| P329R | DB | M | 17 | 6 | 1 | 0 | 0 | China | Yu et al, 2021  (PMID: 33413180) |
| P329L | DB | M | 9 | 3 | 0 | 0 | 0 | Germany | Toubiana et al, 2016  (PMID: 39413163) |
| P329L | DB | F | 21 | NR | 0 | 0 | 0 | USA | Vargas-Hernandez et al, 2018  (PMID: 29111217) |
| Q330K | DB | M | 6 | NR | 0 | 0 | 0 | China | Chen et al, 2019  (PMID: 31686315) |
| G338V | DB | M | 1.5 | 0 | 0 | 1 | 0 | Austria | Fink et al 2024  (PMID: 39114664) |
| G338V | DB | M | 4 | 0 | 0 | 1 | 0 | Austria | Fink et al 2024  (PMID: 39114664) |
| Q340P | DB | F | 33 | NR | 0 | 0 | 0 | USA | Zimmmerman et al, 2017  (PMID: 29226168) |
| K344E | DB | M | 9 | 2 | 1 | 0 | 0 | France | Toubiana et al, 2016  (PMID: 39413163) |
| K344E | DB | F | 5 | 0.5 | 0 | 0 | 0 | India | Staines-Boone et al, 2024  (PMID: 38129739) |
| K344E | DB | F | 4 | NR | 1 | 0 | 0 | Spain | Rudilla et al, 2019  (PMID: 31681265) |
| K344Q | DB | M | 10 | 3 |  | 0 | 0 | UK | Albuquerque et al, 2023  (PMID: 36722341) |
| L351F | DB | M | 9 | NR | 0 | 0 | 0 | China | Chen et al, 2019  (PMID: 31686315) |
| L351F | DB | F | 21 | 1.5 | 1 | 0 | 0 | France | Toubiana et al, 2016  (PMID: 39413163) |
| L351F | DB | F | 8 | 0.42 | 0 | 0 | 0 | Italy | Dotta et al, 2016  (PMID: 26732859) |
| L351F | DB | M | 33 | 0.5 | 0 | 0 | 0 | Italy | Dotta et al, 2016  (PMID: 26732859) |
| L351F | DB | F | 11 | 3 | 0 | 0 | 0 | Spain | Hernandez et al, 2021  (PMID: 34109505) |
| L351F | DB | M | 11 | NR | 0 | 0 | 0 | USA | Forbes et al, 2018  (PMID: 30092289) |
| L351F | DB | M | 11 | 4 | 0 | 0 | 0 | USA | Largent et al, 2023  (PMID: 37406138) |
| E353K | DB | M | 10 | 3 | 0 | 1 | 0 | France | Toubiana et al, 2016  (PMID: 39413163) |
| E353K | DB | F | 43 | NR | 0 | 1 | 0 | France | Toubiana et al, 2016  (PMID: 39413163) |
| E353K | DB | F | 22 | NP | 0 | 0 | 0 | USA | Vargas-Hernandez et al, 2018  (PMID: 29111217) |
| E353K | DB | F | 25 | NR | 0 | 0 | 0 | USA | Toubiana et al, 2016  (PMID: 39413163) |
| E353K | DB | F | 27 | NR | 0 | 0 | 0 | USA | Vargas-Hernandez et al, 2018  (PMID: 29111217) |
| E353K | DB | M | 27 | 14 | 0 | 0 | 0 | USA | Zimmerman et al, 2019  (PMID: 31354696) |
| L354M | DB | M | 9 | 2 | 0 | 1 | 0 | Japan | Toubiana et al, 2016  (PMID: 39413163) |
| L354M | DB | M | 48 | 2 | 0 | 1 | 0 | Japan | Toubiana et al, 2016  (PMID: 39413163) |
| L354V | DB | M | 30 | NR | 1 | 0 | 0 | Spain | Rudilla et al, 2019  (PMID: 31681265) |
| N355D | DB | M | 24 | 0.08 | 0 | 1 | 0 | France | Toubiana et al, 2016  (PMID: 39413163) |
| N355D | DB | M | 28 | 1 | 0 | 1 | 0 | France | Toubiana et al, 2016  (PMID: 39413163) |
| N355D | DB | M | 51 | 0.08 | 0 | 1 | 0 | France | Toubiana et al, 2016  (PMID: 39413163) |
| N357D | DB | M | 6 | 2.5 | 0 | 0 | 0 | China | Wang et al, 2016  (PMID: 27808400) |
| N357D | DB | M | 8 | NR | 0 | 1 | 0 | Czech Republic | Parackova et al., 2023  (PMID: 37358695) |
| N357D | DB | F | 11 | NR | 1 | 0 | 0 | Czech Republic | Parackova et al., 2023  (PMID: 37358695) |
| N357D | DB | M | 45 | NR | 1 | 0 | 0 | Czech Republic | Parackova et al., 2023  (PMID: 37358695) |
| N357D | DB | F | 26 | 10 | 0 | 0 | 0 | Germany | Toubiana et al, 2016  (PMID: 39413163) |
| N357D | DB | F | 27 | 3 | 0 | 0 | 0 | Germany | Toubiana et al, 2016  (PMID: 39413163) |
| L358F | DB | F | 16 | 5 | 0 | 0 | 0 | China | Lee et al, 2019  (PMID: 31572394) |
| L358F | DB | F | 21 | 4 | 0 | 0 | 0 | China | Liu et al, 2024  (PMID: 38072195) |
| L358W | DB | M | 4 | 2 | 0 | 0 | 0 | USA | Bierman-Chow et al, 2022  (PMID: 35435464) |
| L358W | DB | M | 4 | NP | 0 | 0 | 0 | USA | Toubiana et al, 2016  (PMID: 39413163) |
| E370D | DB | M | 26 | 24 | 0 | 0 | 0 | USA | Toubiana et al, 2016  (PMID: 39413163) |
| G384C | DB | M | 1 | 1 | 1 | 0 | 0 | Algeria | Toubiana et al, 2016  (PMID: 39413163) |
| G384D | DB | M | 24 | 3 | 0 | 0 | 0 | China | Li et al 2018  (PMID: 30100585) |
| G384D | DB | M | 5 | 1 | 0 | 0 | 0 | Japan | Yamazaki et al, 2014  (PMID: 25288569) |
| G384D | DB | F | 35 | 19 | 0 | 0 | 0 | Japan | Yamazaki et al, 2014  (PMID: 25288569) |
| G384D | DB | M | 1 | 0.1 | 0 | 0 | 0 | Peru | Toubiana et al, 2016  (PMID: 39413163) |
| G384D | DB | M | 3 | 0.1 | 0 | 1 | 0 | UK | Sanghvi et al, 2020  (PMID: 32349899) |
| T385M | DB | M | 5 | 0.5 | 0 | 0 | 0 | Algeria | Toubiana et al, 2016  (PMID: 39413163) |
| T385M | DB | M | 15 | 0.33 | 0 | 0 | 0 | Argentina | Toubiana et al, 2016  (PMID: 39413163) |
| T385M | DB | M | 22 | 0.1 | 0 | 0 | 0 | Belgium | Toubiana et al, 2016  (PMID: 39413163) |
| T385M | DB | F | 15 | 0.17 | 0 | 0 | 0 | Brazil | Toubiana et al, 2016  (PMID: 39413163) |
| T385M | DB | M | 10 | NR | 0 | 0 | 0 | Canada | Leiding et al, 2018  (PMID: 28601685) |
| T385M | DB | M | 20 | 2 | 0 | 0 | 0 | Canada | Wessel et al 2015  (DOI: https://doi.org/10.14785/lpsn-2015-0013) |
| T385M | DB | F | 32 | 3 | 0 | 0 | 0 | Canada | Wessel et al 2015  (https://doi.org/10.14785/lpsn-2015-0013) |
| T385M | DB | F | 7 | NR | 0 | 0 | 0 | Canada | Leiding et al, 2018  (PMID: 28601685) |
| T385M | DB | F | 1 | 1 | 1 | 0 | 0 | China | Lanqin et al, 2021  (PMID: 34333925) |
| T385M | DB | M | 2 | 0.5 | 1 | 0 | 0 | China | Liu et al 2020  (PMID: 33344614) |
| T385M | DB | F | 4 | NR | 0 | 0 | 0 | China | Chen et al, 2019  (PMID: 31686315) |
| T385M | DB | F | 8 | NR | 1 | 0 | 0 | China | Chen et al, 2024  (PMID: 38758476) |
| T385M | DB | M | 1.42 | 0.1 | 1 | 0 | 0 | China | Ruan et al, 2023  (PMID: 36881481) |
| T385M | DB | M | 7 | 6 | 1 | 0 | 0 | China | Lanqin et al, 2021  (PMID: 34333925) |
| T385M | DB | F | 2 | 0.5 | 1 | 0 | 0 | France | Renoux et al, 2020  (PMID: 31767209) |
| T385M | DB | F | 6 | 2.5 | 0 | 0 | 0 | France | Toubiana et al, 2016  (PMID: 39413163) |
| T385M | DB | M | 9 | 0.33 | 0 | 0 | 0 | France | Toubiana et al, 2016  (PMID: 39413163) |
| T385M | DB | F | 45 | 0.25 | 0 | 0 | 0 | France | Toubiana et al, 2016  (PMID: 39413163) |
| T385M | DB | M | 5 | 2 | 0 | 0 | 0 | India | Toubiana et al, 2016  (PMID: 39413163) |
| T385M | DB | M | 1 | 0.1 | 0 | 1 | 0 | Iran | Toubiana et al, 2016  (PMID: 39413163) |
| T385M | DB | M | 32 | 0.1 | 0 | 1 | 0 | Iran | Toubiana et al, 2016  (PMID: 39413163) |
| T385M | DB | M | 10 | 0.17 | 0 | 0 | 0 | Italy | Dotta et al, 2016  (PMID: 26732859) |
| T385M | DB | M | 15 | 5 | 0 | 0 | 0 | Italy | Dotta et al, 2016  (PMID: 26732859) |
| T385M | DB | M | 0.17 | 0.17 | 0 | 0 | 0 | Japan | Moriya et al, 2020  (PMID: 32180118) |
| T385M | DB | F | 19 | 0.66 | 1 | 0 | 0 | Korea | Lee et al, 2020  (PMID: 31805313) |
| T385M | DB | F | 9 | 0.66 | 1 | 0 | 0 | Mexico | Pedraza-Sanchez et al 2017  (PMID: 29270166) |
| T385M | DB | F | 14 | 1 | 0 | 0 | 0 | Poland | Toubiana et al, 2016  (PMID: 39413163) |
| T385M | DB | M | NR | NR | 0 | 0 | 0 | Russia | Leiding et al, 2018  (PMID: 28601685) |
| T385M | DB | F | 8 | 1.5 | 0 | 0 | 0 | Saudi Arabia | Alakeel et al, 2024  (PMID: 39687689) |
| T385M | DB | F | 8 | 2 | 0 | 0 | 0 | Saudi Arabia | Alidrisi et al, 2022  (PMID: 35498362) |
| T385M | DB | M | NR | NR | 0 | 0 | 0 | Spain | Leiding et al, 2018  (PMID: 28601685) |
| T385M | DB | F | 3 | 2.2 | 0 | 0 | 1 | Turkey | Akarcan et al 2017  (PMID: 29259832) |
| T385M | DB | M | 7 | 0.5 | 0 | 0 | 0 | Turkey | Toubiana et al, 2016  (PMID: 39413163) |
| T385M | DB | M | 8 | 1 | 0 | 0 | 1 | Turkey | Toubiana et al, 2016  (PMID: 39413163) |
| T385M | DB | F | 3.5 | 0.83 | 1 | 0 | 0 | Turkey | Kayaoglu et al, 2021  (PMID: 33475942) |
| T385M | DB | F | 15 | 2 |  | 0 | 0 | UK | Albuquerque et al, 2023  (PMID: 36722341) |
| T385M | DB | M | 15 | 0.1 | 0 | 0 | 0 | Ukraine | Toubiana et al, 2016  (PMID: 39413163) |
| T385M | DB | F | 5 | NR | 1 | 0 | 0 | USA | Takeda et al, 2021  (PMID: 33439110) |
| T385M | DB | F | 5 | 1.5 | 1 | 0 | 0 | USA | Toubiana et al, 2016  (PMID: 39413163) |
| T385M | DB | M | 11 | 2 | 0 | 0 | 0 | USA | Bernasconi et al, 2017  (PMID: 30302727) |
| T385M | DB | M | 15 | NR | 0 | 0 | 0 | USA | Vargas-Hernandez et al, 2018  (PMID: 29111217) |
| T385M | DB | F | 16 | NR | 0 | 0 | 0 | USA | Vargas-Hernandez et al, 2018  (PMID: 29111217) |
| T385M | DB | M | 17 | 0.33 | 0 | 0 | 0 | USA | Forbes et al, 2018  (PMID: 30092289) |
| T385M | DB | M | 17 | NR | 0 | 0 | 0 | USA | Marinelli et al, 2020  (PMID: 32327459) |
| T385M | DB | F | 19 | 0.5 | 0 | 0 | 0 | USA | Toubiana et al, 2016  (PMID: 39413163) |
| T385M | DB | M | 20 | NR | 0 | 0 | 0 | USA | Chaimowitz et al, 2020  (PMID: 33027576) |
| T385M | DB | M | 20 | 2 | 0 | 0 | 0 | USA | Zerbe et al 2016  (PMID: 26743090) |
| T385M | DB | M | 26 | 1.33 | 0 | 0 | 0 | USA | Toubiana et al, 2016  (PMID: 39413163) |
| T385M | DB | M | 27 | 0.08 | 0 | 0 | 0 | USA | Zimmerman et al, 2019  (PMID: 31354696) |
| T385M | DB | M | 28 | NR | 0 | 0 | 0 | USA | Vargas-Hernandez et al, 2018  (PMID: 29111217) |
| T385M | DB | M | 30 | NR | 0 | 0 | 0 | USA | Vargas-Hernandez et al, 2018  (PMID: 29111217) |
| T385K | DB | NR | NR | NR | 1 | 0 | 0 | USA | Depner et al, 2016  (PMID: 26604104) |
| T385M | DB | F | 19 | 10 | 0 | 0 | 0 | USA | Bierman-Chow et al, 2022  (PMID: 35435464) |
| T387A | DB | F | 5 | 0.17 | 0 | 0 | 0 | Belgium | Toubiana et al, 2016  (PMID: 39413163) |
| T387A | DB | M | 0.5 | 0.5 | 0 | 1 | 0 | China | Luo et al 2024  (PMID: 39707011) |
| T387A | DB | F | 41 | 2 | 1 | 0 | 0 | China | Luo et al 2024  (PMID: 39707011) |
| T387A | DB | M | 18 | 7 | 1 | 0 | 0 | Italy | Dotta et al, 2016  (PMID: 26732859) |
| K388E | DB | F | 9 | NP | 0 | 0 | 0 | Argentina | Bernasconi et al, 2017  (PMID: 30302727) |
| K388E | DB | F | 8 | 0.5 | 0 | 0 | 0 | Belgium | Toubiana et al, 2016  (PMID: 39413163) |
| K388E | DB | M | 15 | 5 | 0 | 0 | 0 | China | Zhang et al 2020  (PMID: 32046674) |
| K388E | DB | M | 24 | 4 | 0 | 0 | 0 | China | Wang et al, 2016  (PMID: 27808400) |
| K388E | DB | F | 2 | 0.1 | 1 | 0 | 0 | France | Toubiana et al, 2016  (PMID: 39413163) |
| K388E | DB | F | 18 | 0.25 | 0 | 0 | 0 | France | Toubiana et al, 2016  (PMID: 39413163) |
| K388E | DB | M | 4 | 1.8 | 0 | 1 | 0 | Japan | Toubiana et al, 2016  (PMID: 39413163) |
| K388E | DB | M | 33 | 0.25 | 0 | 1 | 0 | Japan | Toubiana et al, 2016  (PMID: 39413163) |
| K388E | DB | F | 6 | 1 | 0 | 1 | 0 | Switzerland | Toubiana et al, 2016  (PMID: 39413163) |
| K388E | DB | M | 36 | 0.33 | 0 | 1 | 0 | Switzerland | Toubiana et al, 2016  (PMID: 39413163) |
| K388E | DB | M | 8 | 2 | 0 | 1 | 0 | Switzerland | Toubiana et al, 2016  (PMID: 39413163) |
| K388E | DB | F | 6 | 0.83 | 0 | 1 | 0 | Thailand | Toubiana et al, 2016  (PMID: 39413163) |
| K388E | DB | F | 11 | 1 | 0 | 1 | 0 | Thailand | Toubiana et al, 2016  (PMID: 39413163) |
| K388E | DB | M | 40 | 0.5 | 0 | 1 | 0 | Thailand | Toubiana et al, 2016  (PMID: 39413163) |
| K388E | DB | F | 40 | 0.5 |  | 0 | 0 | UK | Albuquerque et al, 2023  (PMID: 36722341) |
| V389A | DB | F | 10 | 0.17 | 1 | 0 | 0 | Estonia | Toubiana et al, 2016  (PMID: 39413163) |
| V389L | DB | F | 23 | 6 | 0 | 0 | 0 | USA | Hartono et al 2018  (PMID: 30317461) |
| M390T | DB | M | 8 | NR | 1 | 0 | 0 | Chile | Toubiana et al, 2016  (PMID: 39413163) |
| M390I | DB | M | 9 | NR | 0 | 0 | 0 | China | Lee et al, 2019  (PMID: 31572394) |
| M390I | DB | M | 40 | 6 | 0 | 0 | 0 | China | Lee et al, 2019  (PMID: 31572394) |
| M390T | DB | F | 20 | NR | 1 | 0 | 0 | Czech Republic | Parackova et al., 2023  (PMID: 37358695) |
| M390T | DB | F | 10 | 0.25 | 1 | 0 | 0 | Estonia | Toubiana et al, 2016  (PMID: 39413163) |
| M390I | DB | M | 4 | 0.08 | 0 | 0 | 0 | France | Toubiana et al, 2016  (PMID: 39413163) |
| M390T | DB | F | 5 | NR | 0 | 0 | 0 | Japan | Leiding et al, 2018  (PMID: 28601685) |
| M390T | DB | M | 30 | 2 | 0 | 0 | 0 | Japan | Toubiana et al, 2016  (PMID: 39413163) |
| M392T | DB | M | 13 | 5 | 0 | 1 | 0 | China | Wang et al, 2022  (PMID: 36335528) |
| M392T | DB | M | NR | NR | 0 | 0 | 0 | China | Wang et al, 2022  (PMID: 36335528) |
| M392T | DB | M | 23 | 15 | 1 | 0 | 0 | France | Toubiana et al, 2016  (PMID: 39413163) |
| M392T | DB | F | 38 | 4 | 1 | 0 | 0 | France | Toubiana et al, 2016  (PMID: 39413163) |
| N397D | DB | F | NR | NR | 0 | 0 | 0 | Canada | Leiding et al, 2018  (PMID: 28601685) |
| N397D | DB | F | 2 | 0.08 | 1 | 0 | 0 | Peru | Toubiana et al, 2016  (PMID: 39413163) |
| L400Q | DB | M | 3 | 0.1 | 1 | 0 | 1 | Egypt | Toubiana et al, 2016  (PMID: 39413163) |
| L400V | DB | M | 55 | 1 | 0 | 0 | 0 | Germany | Toubiana et al, 2016  (PMID: 39413163) |
| L400V | DB | F | 7 | 2 | 0 | 0 | 0 | Italy | Dotta et al, 2016  (PMID: 26732859) |
| L400Q | DB | F | 20 | 2 | 0 | 0 | 0 | USA | Zerbe et al 2016  (PMID: 26743090) |
| F404Y | DB | M | 5 | 0.1 | 0 | 1 | 0 | Canada | Toubiana et al, 2016  (PMID: 39413163) |
| F404Y | DB | M | 5 | 0.1 | 0 | 1 | 0 | Canada | Toubiana et al, 2016  (PMID: 39413163) |
| F404Y | DB | M | 34 | 1 | 0 | 1 | 0 | Canada | Toubiana et al, 2016  (PMID: 39413163) |
| F404V | DB | M | 20 | 1 |  | 0 | 0 | UK | Albuquerque et al, 2023  (PMID: 36722341) |
| L407V | DB | F | 4 | 1 | 0 | 1 | 0 | Hungary | Erdos et al, 2020  (PMID: 32547544) |
| L407V | DB | F | 31 | 3 | 1 | 0 | 0 | Hungary | Erdos et al, 2020  (PMID: 32547544) |
| G416R | DB | F | 57 | 0.25 |  | 0 | 0 | UK | Albuquerque et al, 2023  (PMID: 36722341) |
| T419R | DB | M | 33 | 3 | 1 | 0 | 0 | Austria | Schwärzler et al, 2023  (PMID: 37620121) |
| T419K | DB | M | 25 | 23 |  | 0 | 0 | UK | Albuquerque et al, 2023  (PMID: 36722341) |
| T419R | DB | M | 5 | 0.1 | 0 | 1 | 0 | USA | Toubiana et al, 2016  (PMID: 39413163) |
| T419R | DB | F | 24 | 2.5 | 0 | 1 | 0 | USA | Toubiana et al, 2016  (PMID: 39413163) |
| T437I | DB | M | 23 | 1 | 0 | 0 | 0 | China | Wang et al, 2016  (PMID: 27808400) |
| T437N | DB | F | 5 | NP | 0 | 0 | 0 | USA | Henrickson et al 2019  (PMID: 31114772) |
| T437N | DB | M | 12 | NP | 0 | 0 | 0 | USA | Henrickson et al 2019  (PMID: 31114772) |
| S462R | DB | F | 7 | 7 | 0 | 1 | 0 | Israel | Shamriz et al, 2021  (PMID: 34114647) |
| S462R | DB | F | 12 | 9 | 0 | 1 | 0 | Israel | Shamriz et al, 2021  (PMID: 34114647) |
| S462R | DB | M | 15 | 0.58 | 0 | 1 | 0 | Israel | Shamriz et al, 2021  (PMID: 34114647) |
| S462R | DB | M | 46 | 15 | 1 | 0 | 0 | Israel | Shamriz et al, 2021  (PMID: 34114647) |
| S466R | DB | M | 28 | 6 | 0 | 0 | 0 | China | Liu et al, 2024  (PMID: 38072195) |
| S466R | DB | M | 17 | 3 | 0 | 0 | 0 | Italy | Stellacci et al 2019  (PMID: 31448411) |
| S466R | DB | F | 7 | 0.08 | 1 | 0 | 0 | UK | Toubiana et al, 2016  (PMID: 39413163) |
| S466R | DB | M | 8 | 2 | 1 | 0 | 0 | USA | Eng et al, 2020  (PMID: 32629017) |
| W468R | DB | F | 38 | 3.5 | 0 |  | 0 | Taiwan | Lei et al, 2024  (PMID: 39177867) |
| D517G | L | M | 12 | 3 | 1 | 0 | 0 | Israel | Toubiana et al, 2016  (PMID: 39413163) |
| C543R | L | F | 0.58 | 0.04 | 1 | 0 | 0 | Morocco | Acker et al, 2020  (PMID: 32732635) |
| C543R | L | F | 1 | NR | 0 | 0 | 0 | USA | Forbes et al, 2018  (PMID: 30092289) |
| E545K | L | F | 10 | 6 | 0 | 0 | 0 | USA | Weinacht et al 2017  (PMID: 28139313) |
| E559G | L | F | 5 | 1 | 1 | 0 | 0 | USA | Aluri et al, 2023  (PMID: 37188830) |
| E563Q | L | F | 4 | 0.25 |  | 0 | 0 | Mexico | Staines-Boone et al, 2024  (PMID: 38129739) |
| N574I | L | F | 33 | 0.75 | 0 | 0 | 0 | Belgium | Toubiana et al, 2016  (PMID: 39413163) |
| N574H | L | M | 11 | 1.25 | 0 | 0 | 0 | Ireland | Dhanhani et al 2017  (PMID: 30054782) |
| E609K | SH2 | M | 10 | 1 | 0 | 0 | 0 | USA | Largent et al, 2023  (PMID: 37406138) |
| E609K | SH2 | M | 12 | NR | 0 | 0 | 0 | USA | Forbes et al, 2018  (PMID: 30092289) |
| H629Y | SH2 | F | 30 | 1 | 0 | 0 | 0 | UK | Ovadia et al 2018  (PMID: 30131873) |
| H629Y | SH2 | F | 25 | 5 | 0 | 0 | 0 | USA | Sobh et al 2016  (PMID: 26948078) |
| V653I | SH2 | F | 24 | 6 | 0 | 1 | 0 | Netherlands | Meesilpavikkai et al, 2017  (PMID: 28348565) |
| V653I | SH2 | M | 50 | NR | 0 | 0 | 0 | Netherlands | Meesilpavikkai et al, 2017  (PMID: 28348565) |
| N658S | SH2 | F | 29 | 0.1 | 1 | 0 | 0 | Spain | Toubiana et al, 2016  (PMID: 39413163) |
| E705Q | TAD | M | 10 | NR | 0 | 0 | 0 | Ireland | Shehri et al 2019  (PMID: 31512162) |
| E705V | TAD | M | 36 | 34 | 0 | 1 | 0 | USA | Sampaio et al 2018  (PMID: 28859974) |
| S708F | TAD | M | 1 | NR | 0 | 0 | 0 | China | Chen et al, 2019  (PMID: 31686315) |
| E711Q | TAD | M | 17 | NR | 0 | 1 | 0 | Mexico | Saez de Ocariz et al, 2020  (PMID: 31991004) |
| E711Q | TAD | F | 19 | 15 | 0 | 1 | 0 | Mexico | Toubiana et al, 2016  (PMID: 39413163) |
| E711Q | TAD | F | 23 | NR | 0 | 1 | 0 | Mexico | Saez de Ocariz et al, 2020  (PMID: 31991004) |
| E711Q | TAD | F | 28 | 10 | 0 | 1 | 0 | Mexico | Saez de Ocariz et al, 2020  (PMID: 31991004) |
| E711Q | TAD | F | 45 | NR | 0 | 1 | 0 | Mexico | Saez de Ocariz et al, 2020  (PMID: 31991004) |
| E711Q | TAD | M | 52 | NR | 0 | 1 | 0 | Mexico | Saez de Ocariz et al, 2020  (PMID: 31991004) |
| E711Q | TAD | F | 54 | NR | 0 | 1 | 0 | Mexico | Saez de Ocariz et al, 2020  (PMID: 31991004) |
| E711Q | TAD | M | 85 | NR | 0 | 1 | 0 | Mexico | Saez de Ocariz et al, 2020  (PMID: 31991004) |
| T720I | TAD | M | 21 | 16 | 0 | 0 | 0 | Canada | Toubiana et al, 2016  (PMID: 39413163) |
| T720I | TAD | M | 61 | 15 |  | 0 | 0 | UK | Albuquerque et al, 2023  (PMID: 36722341) |
| T720I | TAD | M | 66 | 5 | 0 | 0 | 0 | UK | Carey et al, 2019  (PMID: 29702748) |
| T720I | TAD | M | 27 | 16 | 0 | 0 | 0 | USA | Bierman-Chow et al, 2022  (PMID: 35435464) |
| P725L | TAD | M | 13 | 2 | 0 | 1 | 0 | Taiwan | Lei et al, 2024  (PMID: 39177867) |
| P725L | TAD | F | 20 | 3 | 0 | 1 | 0 | Taiwan | Lei et al, 2024  (PMID: 39177867) |
| P725L | TAD | M | 22 | 2 | 0 | 1 | 0 | Taiwan | Lei et al, 2024  (PMID: 39177867) |
| P725L | TAD | M | 46 | 2 | 0 | 1 | 0 | Taiwan | Lei et al, 2024  (PMID: 39177867) |
