# Supplementary material for: The T385M STAT1 gain-of-function mutation confers the most severe disease outcomes
Source: Front Immunol. 2025 Nov 28;16:1717692. doi: 10.3389/fimmu.2025.1717692 (PMC12698592; doi:10.3389/fimmu.2025.1717692)
Supplement: Supplementary file 2 [file Table2.docx]

**Table S2: Infections associated with STAT1 GOF PID.**

1 indicates the clinical complication was observed.

| Mutation | CMC | Dermatophytic | Systemic fungal | Bacterial | Mycobacterial | Viral |
| --- | --- | --- | --- | --- | --- | --- |
| E29A | 1 | 0 | 0 | 1 | 0 | 0 |
| D65N | 1 | 1 | 0 | 1 | 0 | 0 |
| D65N | 1 | 1 | 0 | 0 | 0 | 0 |
| D65N | 1 | 1 | 0 | 0 | 0 | 0 |
| D65N | 1 | 0 | 1 | 0 | 0 | 0 |
| Q67R | 1 | 1 | 0 | 0 | 0 | 0 |
| Y68C | 1 | 0 | 0 | 1 | 0 | 0 |
| Y68C | 1 | 0 | 0 | 1 | 0 | 0 |
| S69R | 1 | 1 | 0 | 1 | 0 | 1 |
| R70P | 1 | 1 | 0 | 0 | 0 | 0 |
| R70H | 1 | 0 | 0 | 1 | 0 | 1 |
| N89Y | 1 | 0 | 0 | 1 | 0 | 0 |
| T133A | 1 | 0 | 0 | 0 | 0 | 0 |
| D151E | 1 | 0 | 0 | 1 | 0 | 0 |
| D151E | 1 | 0 | 0 | 0 | 0 | 0 |
| I156T | 1 | 0 | 0 | 1 | 0 | 1 |
| I156T | 1 | 0 | 0 | 1 | 0 | 1 |
| I156T | 1 | 0 | 0 | 1 | 0 | 0 |
| I156T | 0 | 0 | 0 | 1 | 0 | 0 |
| I156T | 1 | 0 | 0 | 1 | 0 | 0 |
| I156T | 0 | 0 | 0 | 1 | 0 | 0 |
| I156T | 0 | 0 | 0 | 1 | 0 | 0 |
| I160F | 1 | 0 | 0 | 1 | 0 | 0 |
| L163R | 1 | 0 | 0 | 1 | 0 | 1 |
| D165H | 1 | 1 | 0 | 1 | 0 | 1 |
| D165H | 1 | 0 | 0 | 1 | 0 | 1 |
| D165G | 1 | 0 | 1 | 0 | 0 | 0 |
| D165G | 1 | 1 | 0 | 1 | 0 | 0 |
| D165H | 1 | 0 | 0 | 1 | 0 | 1 |
| Q167H | 1 | 0 | 0 | 1 | 1 | 0 |
| Q167H | 1 | 0 | 0 | 1 | 1 | 0 |
| Q167E | 1 | 0 | 0 | 1 | 0 | 0 |
| Q167P | 1 | 0 | 0 | 0 | 0 | 0 |
| Q167P | 1 | 1 | 0 | 1 | 0 | 1 |
| D168E | 1 | 0 | 0 | 1 | 0 | 0 |
| D168E | 1 | 0 | 0 | 1 | 0 | 1 |
| Y170N | 1 | 0 | 0 | 0 | 0 | 0 |
| D171N | 1 | 1 | 0 | 0 | 1 | 1 |
| D171N | 1 | 0 | 0 | 1 | 0 | 0 |
| F172L | 1 | 1 | 0 | 1 | 0 | 0 |
| F172L | 1 | 1 | 1 | 0 | 0 | 0 |
| C174R | 1 | 0 | 0 | 0 | 0 | 0 |
| C174R | 1 | 0 | 0 | 0 | 0 | 1 |
| C174R | 1 | 0 | 0 | 0 | 1 | 1 |
| C174R | 1 | 0 | 0 | 0 | 0 | 1 |
| C174R | 1 | 0 | 0 | 1 | 0 | 0 |
| C174R | 1 | 0 | 0 | 1 | 0 | 0 |
| C174R | 1 | 0 | 0 | 0 | 0 | 0 |
| C174R | 1 | 0 | 0 | 1 | 0 | 0 |
| N179K | 1 | 0 | 1 | 1 | 0 | 1 |
| M202V | 1 | 0 | 0 | 0 | 0 | 0 |
| M202T | 1 | 0 | 0 | 1 | 0 | 0 |
| M202I | 1 | 0 | 0 | 1 | 0 | 1 |
| M202I | 1 | 0 | 0 | 0 | 0 | 0 |
| M202V | 1 | 0 | 0 | 1 | 0 | 0 |
| M202V | 1 | 0 | 0 | 1 | 0 | 0 |
| M202V | 1 | 1 | 0 | 1 | 0 | 0 |
| M202V | 1 | 0 | 0 | 1 | 0 | 1 |
| M202I | 1 | 0 | 0 | 1 | 0 | 1 |
| M202V | 1 | 0 | 0 | 1 | 0 | 0 |
| M202T | 1 | 0 | 1 | 1 | 0 | 0 |
| M202V | 1 | 1 | 0 | 1 | 0 | 1 |
| M202I | 1 | 0 | 0 | 1 | 0 | 0 |
| M202V | 1 | 1 | 0 | 0 | 0 | 1 |
| M202I | 1 | 0 | 1 | 0 | 1 | 0 |
| L206P | 1 | 1 | 0 | 1 | 0 | 0 |
| L206H | 1 | 0 | 0 | 0 | 0 | 0 |
| R210G | 1 | 1 | 0 | 1 | 0 | 1 |
| R210K | 1 | 0 | 0 | 1 | 0 | 1 |
| R210K | 1 | 0 | 1 | 0 | 1 | 0 |
| R210I | 1 | 0 | 1 | 1 | 1 | 0 |
| E235A | 1 | 0 | 0 | 1 | 0 | 0 |
| E235G | 1 | 0 | 0 | 1 | 0 | 0 |
| E235A | 1 | 1 | 0 | 1 | 0 | 1 |
| E235G | 1 | 1 | 0 | 1 | 0 | 1 |
| A267E | 0 | 0 | 1 | 1 | 1 | 0 |
| A267V | 1 | 1 | 0 | 0 | 0 | 0 |
| A267V | 1 | 1 | 0 | 0 | 0 | 0 |
| A267V | 1 | 0 | 0 | 1 | 0 | 1 |
| A267T | 1 | 0 | 0 | 0 | 0 | 0 |
| A267T | 1 | 0 | 0 | 0 | 0 | 0 |
| A267T | 1 | 0 | 0 | 0 | 0 | 0 |
| A267T | 1 | 0 | 0 | 0 | 0 | 0 |
| A267V | 1 | 0 | 0 | 0 | 0 | 1 |
| A267V | 1 | 1 | 0 | 1 | 0 | 1 |
| A267V | 1 | 0 | 0 | 0 | 0 | 1 |
| A267V | 1 | 0 | 0 | 1 | 0 | 0 |
| A267V | 1 | 0 | 0 | 0 | 0 | 1 |
| A267V | 1 | 0 | 0 | 1 | 0 | 1 |
| A267V | 1 | 0 | 0 | 0 | 0 | 0 |
| A267V | 1 | 0 | 0 | 0 | 0 | 0 |
| A267V | 1 | 0 | 0 | 0 | 0 | 0 |
| A267V | 1 | 0 | 0 | 1 | 0 | 1 |
| A267V | 1 | 0 | 0 | 1 | 0 | 0 |
| A267V | 1 | 0 | 0 | 1 | 0 | 1 |
| A267V | 1 | 0 | 0 | 1 | 0 | 0 |
| A267V | 1 | 0 | 0 | 1 | 0 | 0 |
| A267V | 1 | 0 | 0 | 1 | 0 | 0 |
| A267V | 1 | 0 | 0 | 1 | 0 | 1 |
| A267V | 1 | 0 | 0 | 1 | 0 | 0 |
| A267V | 1 | 0 | 0 | 0 | 0 | 0 |
| A267V | 1 | 0 | 0 | 0 | 0 | 0 |
| A267V | 1 | 1 | 0 | 0 | 0 | 0 |
| A267V | 1 | 0 | 0 | 1 | 0 | 0 |
| A267V | 1 | 0 | 1 | 1 | 0 | 1 |
| A267V | 1 | 0 | 0 | 0 | 0 | 0 |
| A267V | 1 | 0 | 0 | 1 | 0 | 0 |
| A267V | 1 | 1 | 0 | 1 | 0 | 1 |
| A267V | 1 | 1 | 0 | 1 | 1 | 0 |
| A267V | 1 | 0 | 0 | 1 | 0 | 0 |
| A267V | 1 | 0 | 0 | 0 | 0 | 0 |
| A267V | 1 | 0 | 0 | 0 | 0 | 0 |
| A267V | 1 | 0 | 0 | 0 | 0 | 0 |
| A267V | 1 | 0 | 0 | 1 | 0 | 0 |
| A267V | 1 | 1 | 0 | 0 | 0 | 1 |
| A267V | 1 | 0 | 0 | 1 | 0 | 0 |
| A267V | 1 | 1 | 0 | 1 | 0 | 0 |
| A267V | 1 | 1 | 0 | 1 | 0 | 1 |
| A267V | 1 | 0 | 0 | 1 | 0 | 0 |
| A267V | 1 | 0 | 0 | 0 | 0 | 0 |
| A267V | 1 | 0 | 0 | 0 | 0 | 0 |
| A267V | 1 | 1 | 0 | 0 | 0 | 0 |
| A267V | 1 | 0 | 0 | 1 | 0 | 0 |
| A267V | 1 | 0 | 0 | 1 | 0 | 0 |
| A267V | 1 | 1 | 0 | 1 | 0 | 0 |
| A267V | 1 | 1 | 0 | 1 | 0 | 0 |
| A267V | 1 | 0 | 0 | 1 | 0 | 0 |
| A267V | 1 | 1 | 0 | 1 | 0 | 0 |
| A267V | 1 | 0 | 0 | 1 | 0 | 0 |
| A267V | 1 | 0 | 0 | 1 | 0 | 0 |
| A267V | 1 | 0 | 0 | 0 | 0 | 0 |
| A267V | 1 | 1 | 0 | 1 | 0 | 0 |
| A267V | 1 | 1 | 0 | 1 | 0 | 0 |
| A267V | 1 | 0 | 0 | 1 | 0 | 0 |
| A267V | 0 | 0 | 1 | 0 | 0 | 0 |
| A267V | 0 | 0 | 1 | 1 | 0 | 0 |
| A267V | 1 | 0 | 0 | 0 | 0 | 0 |
| A267V | 1 | 0 | 0 | 0 | 1 | 1 |
| A267V | 1 | 1 | 0 | 1 | 0 | 1 |
| A267V | 1 | 0 | 0 | 1 | 0 | 1 |
| A267V | 1 | 1 | 0 | 0 | 0 | 1 |
| A267V | 1 | 0 | 0 | 1 | 0 | 1 |
| A267V | 1 | 0 | 0 | 1 | 0 | 1 |
| Q271P | 1 | 0 | 0 | 1 | 0 | 1 |
| Q271P | 1 | 1 | 0 | 0 | 0 | 1 |
| Q271P | 1 | 0 | 0 | 0 | 0 | 0 |
| Q271P | 1 | 0 | 0 | 1 | 0 | 0 |
| Q271P | 1 | 1 | 0 | 1 | 0 | 0 |
| R274Q | 1 | 0 | 0 | 0 | 0 | 0 |
| R274W | 1 | 0 | 0 | 0 | 0 | 1 |
| R274W | 1 | 0 | 0 | 0 | 0 | 0 |
| R274W | 1 | 0 | 1 | 1 | 0 | 0 |
| R274W | 1 | 0 | 0 | 1 | 1 | 0 |
| R274Q | 1 | 1 | 0 | 0 | 1 | 0 |
| R274Q | 0 | 0 | 0 | 1 | 0 | 0 |
| R274Q | 1 | 1 | 0 | 0 | 1 | 0 |
| R274Q | 1 | 1 | 0 | 1 | 0 | 1 |
| R274W | 1 | 0 | 0 | 1 | 0 | 1 |
| R274Q | 1 | 0 | 1 | 0 | 1 | 1 |
| R274Q | 1 | 0 | 0 | 0 | 0 | 0 |
| R274Q | 1 | 0 | 0 | 0 | 0 | 0 |
| R274Q | 1 | 0 | 0 | 1 | 0 | 0 |
| R274Q | 1 | 0 | 0 | 0 | 0 | 0 |
| R274Q | 1 | 0 | 0 | 1 | 0 | 0 |
| R274Q | 1 | 0 | 0 | 1 | 0 | 0 |
| R274W | 1 | 0 | 0 | 0 | 0 | 0 |
| R274Q | 1 | 1 | 0 | 1 | 0 | 1 |
| R274W | 1 | 0 | 0 | 1 | 0 | 1 |
| R274Q | 1 | 0 | 1 | 1 | 0 | 0 |
| R274W | 1 | 0 | 0 | 0 | 0 | 0 |
| R274W | 1 | 1 | 1 | 1 | 0 | 0 |
| R274Q | 1 | 0 | 0 | 1 | 0 | 0 |
| R274W | 1 | 0 | 1 | 1 | 0 | 1 |
| R274Q | 1 | 0 | 0 | 1 | 0 | 0 |
| R274Q | 1 | 0 | 0 | 0 | 0 | 0 |
| R274Q | 1 | 0 | 0 | 1 | 0 | 1 |
| R274Q | 1 | 0 | 0 | 0 | 0 | 0 |
| R274Q | 1 | 1 | 0 | 1 | 0 | 1 |
| R274Q | 1 | 0 | 0 | 0 | 0 | 0 |
| R274Q | 1 | 1 | 0 | 1 | 0 | 0 |
| R274W | 1 | 1 | 0 | 1 | 0 | 0 |
| R274W | 1 | 0 | 0 | 1 | 1 | 0 |
| R274W | 1 | 0 | 0 | 1 | 0 | 0 |
| R274Q | 1 | 1 | 0 | 1 | 0 | 0 |
| R274Q | 1 | 0 | 0 | 0 | 0 | 0 |
| R274W | 1 | 0 | 0 | 1 | 0 | 0 |
| R274W | 1 | 0 | 0 | 1 | 0 | 0 |
| R274Q | 1 | 0 | 0 | 1 | 0 | 0 |
| R274Q | 1 | 0 | 1 | 0 | 0 | 0 |
| R274Q | 1 | 0 | 0 | 1 | 0 | 0 |
| R274Q | 1 | 0 | 0 | 1 | 0 | 1 |
| R274W | 1 | 0 | 0 | 0 | 0 | 1 |
| R274W | 1 | 0 | 0 | 1 | 0 | 1 |
| R274Q | 1 | 1 | 0 | 1 | 0 | 0 |
| R274Q | 1 | 0 | 0 | 0 | 0 | 0 |
| R274Q | 1 | 0 | 0 | 0 | 0 | 0 |
| R274Q | 1 | 1 | 0 | 1 | 0 | 0 |
| R274Q | 1 | 1 | 0 | 1 | 1 | 0 |
| R274Q | 0 | 1 | 0 | 1 | 0 | 0 |
| R274Q | 1 | 0 | 0 | 0 | 0 | 0 |
| R274W | 1 | 0 | 0 | 1 | 0 | 1 |
| R274W | 1 | 0 | 1 | 0 | 0 | 0 |
| R274Q | 1 | 0 | 1 | 1 | 0 | 1 |
| R274W | 1 | 1 | 0 | 0 | 0 | 1 |
| R274Q | 1 | 0 | 0 | 0 | 0 | 0 |
| R274Q | 1 | 0 | 0 | 1 | 0 | 0 |
| R274Q | 1 | 0 | 0 | 0 | 0 | 0 |
| R274Q | 1 | 0 | 0 | 0 | 0 | 0 |
| R274Q | 1 | 0 | 0 | 0 | 0 | 0 |
| R274Q | 1 | 1 | 0 | 1 | 0 | 0 |
| R274Q | 1 | 1 | 0 | 1 | 0 | 0 |
| R274Q | 1 | 0 | 0 | 0 | 0 | 0 |
| R274Q | 1 | 0 | 0 | 1 | 0 | 0 |
| R274Q | 1 | 1 | 1 | 1 | 0 | 1 |
| R274W | 1 | 0 | 0 | 0 | 0 | 0 |
| R274W | 1 | 0 | 0 | 1 | 0 | 0 |
| R274W | 1 | 0 | 0 | 1 | 0 | 0 |
| R274W | 1 | 0 | 0 | 0 | 0 | 0 |
| R274W | 1 | 0 | 0 | 0 | 0 | 0 |
| R274G | 1 | 0 | 0 | 1 | 0 | 0 |
| R274W | 1 | 0 | 0 | 1 | 0 | 0 |
| R274W | 1 | 1 | 0 | 1 | 0 | 1 |
| R274W | 1 | 0 | 0 | 0 | 0 | 0 |
| R274Q | 0 | 0 | 0 | 0 | 0 | 0 |
| R274W | 1 | 0 | 0 | 1 | 0 | 0 |
| R274Q | 1 | 0 | 0 | 1 | 0 | 0 |
| R274Q | 1 | 0 | 0 | 0 | 0 | 0 |
| R274W | 1 | 0 | 0 | 0 | 0 | 0 |
| R274W | 1 | 0 | 1 | 1 | 0 | 1 |
| R274W | 1 | 0 | 0 | 1 | 0 | 1 |
| R274W | 1 | 0 | 0 | 0 | 0 | 0 |
| R274W | 1 | 0 | 0 | 1 | 0 | 1 |
| R274W | 1 | 1 | 0 | 1 | 0 | 0 |
| R274W | 1 | 0 | 0 | 0 | 0 | 0 |
| R274W | 1 | 0 | 0 | 1 | 0 | 0 |
| R274W | 1 | 0 | 0 | 1 | 0 | 0 |
| R274W | 1 | 0 | 0 | 0 | 0 | 0 |
| R274W | 1 | 0 | 0 | 0 | 0 | 0 |
| R274W | 1 | 0 | 0 | 0 | 0 | 1 |
| R274Q | 1 | 0 | 0 | 0 | 0 | 0 |
| R274W | 1 | 0 | 0 | 1 | 0 | 0 |
| R274W | 1 | 0 | 0 | 0 | 0 | 0 |
| R274W | 1 | 0 | 0 | 0 | 0 | 0 |
| R274Q | 1 | 0 | 0 | 1 | 0 | 0 |
| R274Q | 1 | 0 | 0 | 0 | 0 | 0 |
| R274Q | 1 | 1 | 0 | 1 | 0 | 1 |
| R274W | 1 | 0 | 0 | 1 | 0 | 0 |
| R274W | 1 | 0 | 0 | 1 | 0 | 0 |
| R274Q | 1 | 0 | 0 | 0 | 1 | 0 |
| K278E | 1 | 0 | 0 | 1 | 0 | 1 |
| L280W | 1 | 0 | 0 | 1 | 0 | 0 |
| L283F | 1 | 0 | 0 | 1 | 0 | 1 |
| L283V | 0 | 1 | 0 | 1 | 0 | 1 |
| L283S | 1 | 0 | 0 | 1 | 0 | 0 |
| L283M | 1 | 1 | 0 | 1 | 0 | 0 |
| L283M | 1 | 1 | 0 | 1 | 0 | 0 |
| E284K | 1 | 0 | 0 | 0 | 0 | 0 |
| E284K | 1 | 0 | 0 | 0 | 0 | 1 |
| E284K | 1 | 1 | 0 | 0 | 0 | 0 |
| Q285R | 1 | 0 | 0 | 1 | 0 | 0 |
| Q285R | 1 | 0 | 0 | 0 | 0 | 1 |
| Q285K | 1 | 1 | 1 | 1 | 1 | 1 |
| Q285K | 1 | 0 | 0 | 1 | 1 | 0 |
| K286I | 1 | 0 | 0 | 1 | 0 | 1 |
| K286I | 1 | 0 | 0 | 1 | 0 | 1 |
| K286I | 1 | 0 | 1 | 1 | 0 | 0 |
| Y287D | 1 | 1 | 0 | 0 | 0 | 0 |
| Y287N | 1 | 1 | 1 | 0 | 0 | 0 |
| Y287H | 1 | 1 | 0 | 1 | 0 | 0 |
| Y287D | 1 | 1 | 0 | 1 | 0 | 1 |
| T288I | 1 | 0 | 0 | 0 | 0 | 0 |
| T288A | 1 | 0 | 1 | 1 | 0 | 1 |
| T288N | 1 | 0 | 0 | 1 | 0 | 1 |
| T288P | 1 | 0 | 0 | 1 | 0 | 0 |
| T288A | 1 | 1 | 0 | 1 | 0 | 0 |
| T288A | 1 | 1 | 0 | 1 | 0 | 1 |
| T288A | 0 | 1 | 0 | 0 | 0 | 0 |
| T288A | 1 | 0 | 1 | 1 | 0 | 0 |
| T288A | 1 | 0 | 0 | 1 | 0 | 1 |
| Y289H | 1 | 0 | 0 | 1 | 0 | 1 |
| Y289C | 1 | 1 | 0 | 0 | 0 | 0 |
| Y289C | 1 | 0 | 0 | 1 | 1 | 0 |
| Y289C | 1 | 0 | 0 | 1 | 0 | 0 |
| Y289C | 1 | 0 | 0 | 1 | 0 | 1 |
| Y289C | 1 | 0 | 0 | 1 | 0 | 1 |
| Y289C | 1 | 0 | 0 | 0 | 0 | 0 |
| Y289C | 1 | 0 | 1 | 1 | 0 | 1 |
| Y289C | 1 | 0 | 1 | 0 | 0 | 1 |
| Y289C | 0 | 0 | 0 | 0 | 0 | 1 |
| Y289C | 0 | 0 | 0 | 0 | 0 | 0 |
| Y289C | 1 | 0 | 0 | 0 | 0 | 0 |
| D292N | 1 | 1 | 0 | 1 | 0 | 1 |
| D292E | 1 | 0 | 0 | 1 | 0 | 1 |
| P293L | 1 | 1 | 0 | 1 | 1 | 0 |
| P293L | 1 | 0 | 0 | 1 | 0 | 0 |
| P293S | 1 | 0 | 0 | 1 | 0 | 1 |
| P293S | 1 | 0 | 0 | 1 | 0 | 1 |
| P293S | 1 | 0 | 0 | 1 | 0 | 1 |
| P293T | 1 | 1 | 0 | 1 | 0 | 1 |
| I294T | 1 | 1 | 0 | 1 | 0 | 1 |
| I294T | 1 | 0 | 0 | 1 | 0 | 1 |
| I294T | 1 | 0 | 0 | 1 | 0 | 1 |
| T295K | 1 | 1 | 0 | 1 | 0 | 0 |
| N297del | 1 | 1 | 0 | 1 | 1 | 0 |
| K298N | 1 | 1 | 0 | 1 | 0 | 0 |
| K298N | 1 | 1 | 0 | 1 | 0 | 0 |
| K298N | 1 | 1 | 0 | 0 | 0 | 0 |
| L301del | 1 | 0 | 1 | 1 | 0 | 1 |
| R321S | 1 | 0 | 1 | 1 | 0 | 0 |
| R321S | 1 | 0 | 0 | 1 | 0 | 0 |
| R321G | 1 | 0 | 0 | 1 | 0 | 1 |
| R321G | 1 | 0 | 0 | 1 | 0 | 0 |
| R321G | 1 | 0 | 1 | 1 | 0 | 1 |
| R321S | 1 | 0 | 0 | 0 | 0 | 0 |
| R321S | 1 | 1 | 0 | 1 | 0 | 0 |
| R321S | 1 | 1 | 0 | 1 | 0 | 1 |
| R321S | 0 | 1 | 0 | 1 | 0 | 1 |
| R321S | 1 | 1 | 0 | 1 | 0 | 1 |
| R321S | 1 | 0 | 0 | 1 | 0 | 0 |
| R321S | 1 | 0 | 0 | 1 | 1 | 1 |
| R321S | 1 | 1 | 1 | 1 | 1 | 1 |
| C324R | 0 | 1 | 0 | 1 | 0 | 0 |
| C324R | 1 | 0 | 0 | 1 | 0 | 0 |
| C324R | 1 | 0 | 0 | 0 | 0 | 0 |
| C324R | 1 | 0 | 0 | 1 | 0 | 0 |
| C324R | 1 | 0 | 0 | 1 | 0 | 0 |
| C324R | 1 | 0 | 0 | 1 | 0 | 1 |
| C324R | 1 | 0 | 1 | 1 | 1 | 1 |
| C324R | 1 | 0 | 0 | 1 | 0 | 1 |
| C324F | 1 | 0 | 0 | 1 | 0 | 1 |
| C324Y | 1 | 0 | 0 | 0 | 0 | 0 |
| M325K | 1 | 1 | 1 | 1 | 0 | 1 |
| H328R | 0 | 0 | 0 | 1 | 1 | 1 |
| H328R | 0 | 0 | 0 | 1 | 1 | 0 |
| H328R | 0 | 0 | 0 | 1 | 1 | 1 |
| P329L | 1 | 0 | 0 | 0 | 0 | 0 |
| P329L | 1 | 0 | 0 | 0 | 0 | 0 |
| P329R | 0 | 0 | 0 | 1 | 0 | 1 |
| P329L | 1 | 0 | 0 | 1 | 0 | 1 |
| P329L | 1 | 0 | 0 | 0 | 0 | 1 |
| Q330K | 1 | 0 | 1 | 1 | 0 | 0 |
| G338V | 1 | 0 | 0 | 0 | 0 | 0 |
| G338V | 1 | 1 | 0 | 0 | 0 | 0 |
| Q340P | 1 | 0 | 0 | 1 | 0 | 1 |
| K344E | 1 | 0 | 0 | 1 | 1 | 0 |
| K344E | 1 | 1 | 0 | 0 | 0 | 0 |
| K344E | 1 | 0 | 0 | 0 | 0 | 0 |
| K344Q | 1 | 0 | 0 | 1 | 0 | 0 |
| L351F | 1 | 0 | 0 | 1 | 1 | 1 |
| L351F | 1 | 0 | 0 | 1 | 0 | 0 |
| L351F | 1 | 0 | 0 | 1 | 0 | 1 |
| L351F | 1 | 1 | 0 | 1 | 0 | 1 |
| L351F | 1 | 0 | 0 | 1 | 0 | 0 |
| L351F | 1 | 0 | 0 | 1 | 0 | 0 |
| L351F | 0 | 0 | 0 | 0 | 0 | 0 |
| E353K | 1 | 0 | 0 | 1 | 0 | 1 |
| E353K | 1 | 0 | 0 | 1 | 0 | 1 |
| E353K | 0 | 1 | 1 | 0 | 0 | 1 |
| E353K | 1 | 1 | 1 | 0 | 0 | 1 |
| E353K | 1 | 1 | 0 | 0 | 0 | 0 |
| E353K | 1 | 0 | 0 | 0 | 0 | 0 |
| L354M | 1 | 0 | 0 | 1 | 0 | 0 |
| L354M | 1 | 1 | 1 | 1 | 0 | 1 |
| L354V | 1 | 1 | 0 | 0 | 0 | 0 |
| N355D | 1 | 0 | 0 | 1 | 1 | 1 |
| N355D | 1 | 1 | 0 | 1 | 0 | 1 |
| N355D | 1 | 0 | 0 | 1 | 0 | 0 |
| N357D | 1 | 0 | 0 | 0 | 0 | 0 |
| N357D | 1 | 0 | 0 | 1 | 0 | 0 |
| N357D | 1 | 0 | 0 | 1 | 0 | 1 |
| N357D | 1 | 0 | 0 | 0 | 0 | 1 |
| N357D | 1 | 0 | 0 | 0 | 0 | 0 |
| N357D | 1 | 0 | 0 | 0 | 0 | 0 |
| L358F | 1 | 0 | 1 | 0 | 1 | 0 |
| L358F | 1 | 1 | 1 | 1 | 0 | 1 |
| L358W | 1 | 0 | 0 | 1 | 0 | 1 |
| L358W | 0 | 1 | 0 | 1 | 0 | 1 |
| E370D | 1 | 0 | 1 | 0 | 0 | 0 |
| G384C | 1 | 0 | 0 | 1 | 0 | 1 |
| G384D | 1 | 0 | 1 | 1 | 0 | 1 |
| G384D | 1 | 1 | 0 | 0 | 0 | 1 |
| G384D | 1 | 1 | 0 | 0 | 0 | 1 |
| G384D | 1 | 0 | 0 | 1 | 0 | 0 |
| G384D | 1 | 0 | 0 | 1 | 0 | 0 |
| T385M | 1 | 1 | 0 | 1 | 0 | 0 |
| T385M | 1 | 1 | 0 | 1 | 1 | 1 |
| T385M | 1 | 0 | 0 | 1 | 0 | 0 |
| T385M | 1 | 0 | 0 | 1 | 0 | 0 |
| T385M | 1 | 0 | 0 | 1 | 0 | 1 |
| T385M | 1 | 0 | 0 | 1 | 0 | 1 |
| T385M | 1 | 0 | 0 | 1 | 0 | 0 |
| T385M | 1 | 0 | 0 | 1 | 0 | 1 |
| T385M | 1 | 0 | 0 | 0 | 1 | 0 |
| T385M | 1 | 0 | 0 | 0 | 1 | 0 |
| T385M | 1 | 0 | 1 | 1 | 0 | 0 |
| T385M | 1 | 0 | 0 | 1 | 0 | 1 |
| T385M | 1 | 0 | 1 | 0 | 0 | 0 |
| T385M | 1 | 0 | 0 | 1 | 0 | 0 |
| T385M | 1 | 0 | 0 | 1 | 0 | 0 |
| T385M | 1 | 0 | 0 | 1 | 0 | 1 |
| T385M | 1 | 0 | 0 | 1 | 1 | 0 |
| T385M | 1 | 0 | 0 | 1 | 0 | 1 |
| T385M | 1 | 1 | 0 | 1 | 0 | 0 |
| T385M | 1 | 0 | 0 | 1 | 0 | 0 |
| T385M | 1 | 1 | 0 | 1 | 0 | 0 |
| T385M | 1 | 0 | 0 | 1 | 0 | 0 |
| T385M | 1 | 0 | 0 | 1 | 0 | 0 |
| T385M | 0 | 0 | 1 | 0 | 0 | 1 |
| T385M | 1 | 0 | 0 | 1 | 1 | 1 |
| T385M | 1 | 0 | 0 | 1 | 1 | 0 |
| T385M | 1 | 0 | 0 | 1 | 1 | 0 |
| T385M | 1 | 0 | 1 | 1 | 1 | 0 |
| T385M | 1 | 1 | 0 | 0 | 0 | 0 |
| T385M | 1 | 1 | 0 | 1 | 0 | 0 |
| T385M | 1 | 0 | 1 | 1 | 1 | 0 |
| T385M | 1 | 1 | 1 | 1 | 1 | 1 |
| T385M | 1 | 0 | 0 | 0 | 0 | 0 |
| T385M | 1 | 0 | 0 | 1 | 0 | 1 |
| T385M | 1 | 0 | 1 | 1 | 0 | 1 |
| T385M | 1 | 0 | 0 | 0 | 0 | 0 |
| T385M | 1 | 1 | 0 | 1 | 0 | 1 |
| T385M | 1 | 0 | 0 | 1 | 0 | 0 |
| T385M | 1 | 0 | 0 | 1 | 0 | 0 |
| T385M | 1 | 0 | 0 | 1 | 0 | 0 |
| T385M | 1 | 0 | 0 | 1 | 0 | 0 |
| T385M | 1 | 0 | 0 | 0 | 0 | 1 |
| T385M | 1 | 0 | 0 | 1 | 0 | 0 |
| T385M | 1 | 0 | 1 | 1 | 0 | 1 |
| T385M | 1 | 0 | 0 | 1 | 1 | 1 |
| T385M | 1 | 0 | 0 | 1 | 0 | 0 |
| T385M | 1 | 0 | 0 | 1 | 0 | 1 |
| T385M | 1 | 0 | 1 | 1 | 1 | 1 |
| T385M | 1 | 0 | 1 | 0 | 1 | 1 |
| T385M | 1 | 0 | 1 | 0 | 1 | 1 |
| T385M | 1 | 0 | 0 | 1 | 0 | 1 |
| T385K | 1 | 0 | 0 | 0 | 0 | 0 |
| T385M | 1 | 0 | 0 | 0 | 1 | 1 |
| T387A | 1 | 0 | 0 | 1 | 0 | 0 |
| T387A | 1 | 1 | 0 | 0 | 0 | 0 |
| T387A | 1 | 1 | 0 | 0 | 0 | 0 |
| T387A | 1 | 0 | 1 | 1 | 0 | 1 |
| K388E | 0 | 0 | 1 | 0 | 0 | 0 |
| K388E | 1 | 1 | 0 | 1 | 0 | 0 |
| K388E | 1 | 1 | 0 | 0 | 0 | 0 |
| K388E | 1 | 1 | 0 | 0 | 0 | 0 |
| K388E | 1 | 0 | 0 | 0 | 0 | 0 |
| K388E | 1 | 0 | 0 | 1 | 0 | 0 |
| K388E | 1 | 0 | 0 | 0 | 0 | 1 |
| K388E | 1 | 1 | 0 | 1 | 0 | 1 |
| K388E | 1 | 0 | 0 | 1 | 0 | 0 |
| K388E | 1 | 0 | 0 | 1 | 0 | 0 |
| K388E | 1 | 0 | 0 | 1 | 0 | 0 |
| K388E | 1 | 0 | 0 | 0 | 0 | 0 |
| K388E | 1 | 0 | 0 | 0 | 0 | 0 |
| K388E | 1 | 0 | 1 | 0 | 0 | 1 |
| K388E | 1 | 0 | 0 | 0 | 0 | 0 |
| V389A | 1 | 0 | 0 | 1 | 0 | 0 |
| V389L | 1 | 0 | 1 | 1 | 0 | 1 |
| M390T | 1 | 0 | 0 | 1 | 0 | 0 |
| M390I | 1 | 0 | 0 | 1 | 1 | 0 |
| M390I | 1 | 0 | 1 | 0 | 0 | 0 |
| M390T | 1 | 0 | 0 | 1 | 0 | 1 |
| M390T | 1 | 0 | 0 | 0 | 0 | 0 |
| M390I | 1 | 0 | 0 | 1 | 1 | 1 |
| M390T | 1 | 0 | 0 | 1 | 0 | 0 |
| M390T | 1 | 0 | 0 | 1 | 0 | 1 |
| M392T | 1 | 0 | 0 | 0 | 0 | 0 |
| M392T | 1 | 0 | 0 | 0 | 0 | 0 |
| M392T | 1 | 0 | 1 | 1 | 0 | 0 |
| M392T | 1 | 0 | 0 | 1 | 0 | 0 |
| N397D | 1 | 0 | 0 | 1 | 0 | 1 |
| N397D | 1 | 0 | 0 | 1 | 0 | 1 |
| L400Q | 1 | 0 | 0 | 1 | 0 | 1 |
| L400V | 1 | 0 | 0 | 1 | 0 | 1 |
| L400V | 1 | 1 | 0 | 1 | 0 | 0 |
| L400Q | 1 | 0 | 0 | 1 | 1 | 1 |
| F404Y | 1 | 1 | 0 | 1 | 0 | 1 |
| F404Y | 1 | 0 | 0 | 1 | 0 | 0 |
| F404Y | 1 | 0 | 0 | 0 | 0 | 0 |
| F404V | 1 | 0 | 0 | 1 | 0 | 0 |
| L407V | 1 | 0 | 0 | 0 | 0 | 1 |
| L407V | 1 | 1 | 1 | 1 | 0 | 1 |
| G416R | 1 | 0 | 0 | 1 | 0 | 0 |
| T419R | 1 | 0 | 0 | 1 | 1 | 0 |
| T419K | 1 | 0 | 0 | 1 | 0 | 1 |
| T419R | 1 | 0 | 0 | 1 | 0 | 0 |
| T419R | 1 | 0 | 0 | 1 | 0 | 1 |
| T437I | 1 | 0 | 0 | 0 | 0 | 0 |
| T437N | 0 | 0 | 0 | 0 | 0 | 0 |
| T437N | 0 | 1 | 0 | 0 | 0 | 1 |
| S462R | 1 | 1 | 0 | 1 | 0 | 0 |
| S462R | 1 | 1 | 0 | 1 | 0 | 0 |
| S462R | 1 | 1 | 0 | 1 | 0 | 0 |
| S462R | 1 | 1 | 0 | 0 | 0 | 0 |
| S466R | 1 | 0 | 0 | 1 | 1 | 1 |
| S466R | 1 | 1 | 0 | 1 | 0 | 0 |
| S466R | 1 | 1 | 0 | 1 | 0 | 1 |
| S466R | 1 | 0 | 0 | 1 | 0 | 1 |
| W468R | 1 | 1 | 0 | 0 | 0 | 1 |
| D517G | 1 | 1 | 0 | 0 | 0 | 0 |
| C543R | 1 | 0 | 0 | 1 | 0 | 0 |
| C543R | 1 | 1 | 1 | 1 | 0 | 0 |
| E545K | 1 | 0 | 0 | 0 | 0 | 0 |
| E559G | 0 | 0 | 0 | 1 | 0 | 0 |
| E563Q | 1 | 1 | 0 | 1 | 0 | 0 |
| N574I | 1 | 0 | 0 | 1 | 0 | 1 |
| N574H | 1 | 0 | 0 | 1 | 0 | 0 |
| E609K | 1 | 0 | 1 | 1 | 0 | 1 |
| E609K | 1 | 0 | 0 | 1 | 0 | 0 |
| H629Y | 1 | 0 | 0 | 1 | 0 | 0 |
| H629Y | 1 | 0 | 0 | 1 | 0 | 1 |
| V653I | 1 | 0 | 0 | 1 | 0 | 1 |
| V653I | 1 | 0 | 0 | 0 | 0 | 0 |
| N658S | 1 | 0 | 1 | 1 | 0 | 0 |
| E705Q | 1 | 1 | 0 | 1 | 0 | 1 |
| E705V | 1 | 0 | 0 | 1 | 0 | 1 |
| S708F | 1 | 0 | 0 | 1 | 0 | 0 |
| E711Q | 1 | 0 | 0 | 0 | 0 | 0 |
| E711Q | 1 | 0 | 0 | 1 | 1 | 0 |
| E711Q | 1 | 0 | 0 | 1 | 0 | 1 |
| E711Q | 1 | 1 | 0 | 0 | 0 | 0 |
| E711Q | 1 | 0 | 0 | 0 | 1 | 0 |
| E711Q | 1 | 0 | 0 | 0 | 0 | 1 |
| E711Q | 1 | 0 | 0 | 0 | 0 | 0 |
| E711Q | 1 | 0 | 0 | 0 | 1 | 0 |
| T720I | 1 | 0 | 0 | 1 | 0 | 0 |
| T720I | 1 | 0 | 0 | 1 | 0 | 0 |
| T720I | 1 | 1 | 0 | 1 | 0 | 0 |
| T720I | 1 | 0 | 0 | 0 | 0 | 0 |
| P725L | 1 | 1 | 0 | 1 | 0 | 1 |
| P725L | 1 | 0 | 0 | 1 | 0 | 1 |
| P725L | 1 | 1 | 0 | 1 | 0 | 1 |
| P725L | 1 | 1 | 0 | 1 | 0 | 0 |
