# Supplementary material for: The T385M STAT1 gain-of-function mutation confers the most severe disease outcomes
Source: Front Immunol. 2025 Nov 28;16:1717692. doi: 10.3389/fimmu.2025.1717692 (PMC12698592; doi:10.3389/fimmu.2025.1717692)
Supplement: Supplementary file 3 [file Table3.docx]

**Table S3: Non-infectious clinical complications associated with STAT1 GOF PID.**

1 indicates the clinical complication was observed.

Other autoimmune symptoms refers to autoantibody-driven diseases only.

| Mutation | Thyroid dysfunction | Other autoimmune symptoms | Atopy | Aneurysm | Tumour | Bronchiectasis | Death (age) | Cause of death |
| --- | --- | --- | --- | --- | --- | --- | --- | --- |
| E29A | 1 | 1 | 0 | 0 | 0 | 1 | 0 | 0 |
| D65N | 0 | 0 | 0 | 0 | 0 | 0 | 0 | 0 |
| D65N | 0 | 0 | 1 | 0 | 0 | 0 | 0 | 0 |
| D65N | 0 | 0 | 1 | 0 | 0 | 0 | 0 | 0 |
| D65N | 0 | 1 | 0 | 0 | 0 | 0 | 0 | 0 |
| Q67R | 0 | 0 | 0 | 0 | 0 | 0 | 0 | 0 |
| Y68C | 0 | 0 | 0 | 0 | 0 | 0 | 0 | 0 |
| Y68C | 1 | 0 | 1 | 0 | 0 | 0 | 0 | 0 |
| S69R | 0 | 0 | 0 | 0 | 0 | 0 | 0 | 0 |
| R70P | 1 | 0 | 0 | 0 | 0 | 0 | 0 | 0 |
| R70H | 1 | 1 | 0 | 0 | 0 | 0 | 0 | 0 |
| N89Y | 0 | 1 | 0 | 0 | 0 | 0 | 0 | 0 |
| T133A | 0 | 0 | 0 | 0 | 0 | 0 | 0 | 0 |
| D151E | 0 | 1 | 1 | 0 | 0 | 0 | 0 | 0 |
| D151E | 0 | 1 | 1 | 0 | 0 | 0 | 0 | 0 |
| I156T | 1 | 1 | 0 | 0 | 0 | 0 | 0 | 0 |
| I156T | 0 | 0 | 0 | 0 | 0 | 0 | 0 | 0 |
| I156T | 1 | 0 | 0 | 0 | 0 | 0 | 0 | 0 |
| I156T | 1 | 0 | 0 | 0 | 0 | 0 | 0 | 0 |
| I156T | 1 | 1 | 0 | 0 | 0 | 0 | 0 | 0 |
| I156T | 0 | 0 | 0 | 0 | 0 | 0 | 0 | 0 |
| I156T | 1 | 0 | 0 | 0 | 0 | 0 | 0 | 0 |
| I160F | 0 | 1 | 1 | 0 | 0 | 0 | 0 | 0 |
| L163R | 0 | 0 | 0 | 0 | 0 | 0 | 0 | 0 |
| D165H | 0 | 0 | 0 | 0 | 0 | 1 | 0 | 0 |
| D165H | 0 | 0 | 0 | 0 | 0 | 0 | 0 | 0 |
| D165G | 0 | 0 | 0 | 0 | 0 | 0 | 0 | 0 |
| D165G | 0 | 0 | 0 | 0 | 0 | 1 | 1 (21) | Infection |
| D165H | 1 | 1 | 0 | 0 | 0 | 0 | 0 | 0 |
| Q167H | 1 | 0 | 0 | 0 | 0 | 0 | 0 | 0 |
| Q167H | 0 | 0 | 0 | 0 | 0 | 1 | 0 | 0 |
| Q167E | 0 | 0 | 0 | 0 | 1 | 0 | 0 | 0 |
| Q167P | 0 | 0 | 0 | 0 | 0 | 0 | 1 (6) | Hepatitis |
| Q167P | 0 | 0 | 0 | 0 | 0 | 0 | 0 | 0 |
| D168E | 0 | 0 | 0 | 0 | 1 | 0 | 0 | 0 |
| D168E | 0 | 1 | 1 | 0 | 0 | 0 | 0 | 0 |
| Y170N | 1 | 0 | 0 | 0 | 0 | 0 | 0 | 0 |
| D171N | 0 | 0 | 0 | 0 | 0 | 0 | 0 | 0 |
| D171N | 0 | 0 | 0 | 0 | 0 | 1 | 0 | 0 |
| F172L | 0 | 0 | 0 | 0 | 0 | 0 | 0 | 0 |
| F172L | 0 | 0 | 0 | 0 | 0 | 0 | 0 | 0 |
| C174R | 1 | 0 | 1 | 0 | 0 | 0 | 0 | 0 |
| C174R | 0 | 0 | 1 | 0 | 0 | 0 | 0 | 0 |
| C174R | 0 | 0 | 1 | 0 | 0 | 0 | 0 | 0 |
| C174R | 0 | 0 | 0 | 0 | 0 | 0 | 0 | 0 |
| C174R | 1 | 1 | 0 | 0 | 0 | 0 | 0 | 0 |
| C174R | 1 | 0 | 1 | 0 | 0 | 0 | 0 | 0 |
| C174R | 0 | 0 | 0 | 0 | 1 | 0 | 1 (58) | Carcinoma |
| C174R | 0 | 1 | 0 | 0 | 0 | 0 | 0 | 0 |
| N179K | 0 | 1 | 1 | 1 | 0 | 1 | 1 (9) | Intracranial haemorrhage |
| M202V | 1 | 0 | 0 | 1 | 0 | 0 | 0 | 0 |
| M202T | 1 | 0 | 0 | 0 | 0 | 0 | 0 | 0 |
| M202I | 0 | 0 | 0 | 0 | 0 | 0 | 0 | 0 |
| M202I | 0 | 0 | 0 | 0 | 0 | 0 | 0 | 0 |
| M202V | 1 | 1 | 1 | 0 | 0 | 1 | 0 | 0 |
| M202V | 0 | 0 | 0 | 0 | 0 | 1 | 0 | 0 |
| M202V | 0 | 0 | 0 | 0 | 0 | 1 | 0 | 0 |
| M202V | 0 | 0 | 0 | 0 | 0 | 1 | 0 | 0 |
| M202I | 0 | 1 | 0 | 1 | 0 | 1 | 0 | 0 |
| M202V | 0 | 1 | 0 | 0 | 1 | 0 | 1 (55) | Carcinoma |
| M202T | 0 | 1 | 0 | 0 | 0 | 0 | 0 | 0 |
| M202V | 0 | 1 | 0 | 0 | 0 | 0 | 0 | 0 |
| M202I | 0 | 0 | 0 | 0 | 0 | 0 | 0 | 0 |
| M202V | 0 | 1 | 0 | 0 | 0 | 0 | 0 | 0 |
| M202I | 0 | 0 | 0 | 0 | 0 | 0 | 0 | 0 |
| L206P | 0 | 0 | 0 | 0 | 0 | 0 | 0 | 0 |
| L206H | 1 | 1 | 0 | 0 | 0 | 0 | 0 | 0 |
| R210G | 0 | 0 | 0 | 0 | 0 | 0 | 0 | 0 |
| R210K | 0 | 0 | 0 | 0 | 0 | 1 | 0 | 0 |
| R210K | 0 | 0 | 0 | 0 | 0 | 0 | 0 | 0 |
| R210I | 0 | 1 | 0 | 0 | 0 | 0 | 1 (12) | Infection |
| E235A | 0 | 0 | 0 | 0 | 0 | 1 | 0 | 0 |
| E235G | 0 | 0 | 0 | 0 | 0 | 0 | 0 | 0 |
| E235A | 0 | 0 | 0 | 1 | 1 | 0 | 0 | 0 |
| E235G | 0 | 1 | 0 | 0 | 0 | 0 | 0 | 0 |
| A267E | 1 | 0 | 0 | 0 | 0 | 0 | 0 | 0 |
| A267V | 0 | 0 | 0 | 0 | 0 | 0 | 0 | 0 |
| A267V | 1 | 0 | 0 | 0 | 0 | 0 | 0 | 0 |
| A267V | 0 | 0 | 1 | 0 | 0 | 0 | 0 | 0 |
| A267T | 0 | 0 | 0 | 0 | 0 | 0 | 0 | 0 |
| A267T | 0 | 0 | 0 | 0 | 0 | 0 | 0 | 0 |
| A267T | 0 | 0 | 0 | 0 | 0 | 0 | 0 | 0 |
| A267T | 0 | 0 | 0 | 0 | 0 | 0 | 0 | 0 |
| A267V | 0 | 0 | 1 | 0 | 0 | 0 | 0 | 0 |
| A267V | 0 | 0 | 1 | 0 | 0 | 0 | 0 | 0 |
| A267V | 1 | 0 | 0 | 0 | 0 | 0 | 0 | 0 |
| A267V | 1 | 1 | 0 | 0 | 0 | 0 | 0 | 0 |
| A267V | 1 | 0 | 0 | 0 | 0 | 0 | 0 | 0 |
| A267V | 1 | 0 | 0 | 1 | 1 | 1 | 0 | 0 |
| A267V | 0 | 0 | 0 | 0 | 0 | 0 | 0 | 0 |
| A267V | 0 | 0 | 0 | 0 | 0 | 0 | 0 | 0 |
| A267V | 0 | 0 | 0 | 0 | 0 | 0 | 0 | 0 |
| A267V | 0 | 0 | 0 | 0 | 0 | 1 | 0 | 0 |
| A267V | 0 | 0 | 0 | 0 | 0 | 0 | 0 | 0 |
| A267V | 0 | 0 | 0 | 0 | 0 | 1 | 0 | 0 |
| A267V | 1 | 0 | 1 | 0 | 0 | 0 | 0 | 0 |
| A267V | 1 | 0 | 1 | 1 | 0 | 0 | 0 | 0 |
| A267V | 0 | 0 | 0 | 0 | 0 | 0 | 0 | 0 |
| A267V | 0 | 0 | 0 | 0 | 0 | 1 | 0 | 0 |
| A267V | 0 | 0 | 0 | 0 | 0 | 1 | 0 | 0 |
| A267V | 0 | 0 | 0 | 0 | 0 | 0 | 0 | 0 |
| A267V | 0 | 0 | 0 | 0 | 0 | 0 | 0 | 0 |
| A267V | 0 | 0 | 0 | 0 | 0 | 0 | 0 | 0 |
| A267V | 0 | 0 | 0 | 0 | 0 | 0 | 0 | 0 |
| A267V | 0 | 0 | 0 | 0 | 0 | 0 | 0 | 0 |
| A267V | 0 | 0 | 0 | 0 | 0 | 0 | 0 | 0 |
| A267V | 0 | 0 | 0 | 0 | 0 | 0 | 0 | 0 |
| A267V | 1 | 1 | 0 | 0 | 0 | 0 | 0 | 0 |
| A267V | 0 | 0 | 0 | 0 | 0 | 1 | 0 | 0 |
| A267V | 0 | 0 | 0 | 0 | 0 | 0 | 0 | 0 |
| A267V | 0 | 0 | 0 | 0 | 0 | 0 | 0 | 0 |
| A267V | 0 | 0 | 0 | 0 | 1 | 0 | 1 (33) | Carcinoma |
| A267V | 0 | 0 | 1 | 0 | 1 | 0 | 0 | 0 |
| A267V | 1 | 0 | 1 | 0 | 0 | 0 | 0 | 0 |
| A267V | 0 | 0 | 1 | 0 | 0 | 0 | 0 | 0 |
| A267V | 0 | 0 | 0 | 0 | 0 | 0 | 0 | 0 |
| A267V | 1 | 1 | 0 | 0 | 0 | 0 | 0 | 0 |
| A267V | 1 | 1 | 0 | 0 | 0 | 1 | 0 | 0 |
| A267V | 0 | 0 | 0 | 0 | 0 | 0 | 0 | 0 |
| A267V | 0 | 0 | 0 | 0 | 0 | 0 | 0 | 0 |
| A267V | 0 | 0 | 0 | 0 | 0 | 0 | 0 | 0 |
| A267V | 0 | 0 | 0 | 0 | 0 | 1 | 0 | 0 |
| A267V | 0 | 0 | 1 | 0 | 0 | 0 | 0 | 0 |
| A267V | 0 | 0 | 1 | 0 | 0 | 0 | 0 | 0 |
| A267V | 1 | 0 | 0 | 0 | 0 | 0 | 0 | 0 |
| A267V | 0 | 0 | 0 | 0 | 0 | 0 | 0 | 0 |
| A267V | 1 | 0 | 0 | 0 | 0 | 0 | 0 | 0 |
| A267V | 0 | 0 | 0 | 0 | 1 | 1 | 1 (40) | Carcinoma |
| A267V | 0 | 0 | 1 | 0 | 1 | 0 | 1 (40) | Carcinoma |
| A267V | 0 | 0 | 0 | 0 | 0 | 0 | 0 | 0 |
| A267V | 0 | 0 | 0 | 0 | 1 | 0 | 1 (42) | Carcinoma |
| A267V | 0 | 0 | 1 | 0 | 0 | 0 | 0 | 0 |
| A267V | 1 | 0 | 0 | 0 | 1 | 0 | 1 (58) | Carcinoma |
| A267V | 1 | 0 | 1 | 0 | 1 | 0 | 0 | 0 |
| A267V | 0 | 0 | 0 | 0 | 0 | 0 | 0 | 0 |
| A267V | 0 | 0 | 0 | 0 | 0 | 0 | 1 (17) | Infection |
| A267V | 0 | 0 | 0 | 0 | 0 | 0 | 0 | 0 |
| A267V | 0 | 0 | 0 | 0 | 0 | 0 | 0 | 0 |
| A267V | 1 | 0 | 0 | 0 | 0 | 0 | 0 | 0 |
| A267V | 1 | 0 | 0 | 0 | 0 | 0 | 0 | 0 |
| A267V | 1 | 0 | 0 | 0 | 0 | 0 | 0 | 0 |
| A267V | 0 | 0 | 0 | 0 | 0 | 0 | 0 | 0 |
| A267V | 1 | 1 | 0 | 0 | 0 | 0 | 0 | 0 |
| Q271P | 0 | 0 | 0 | 0 | 0 | 1 | 1 (41) | SCC |
| Q271P | 1 | 0 | 0 | 0 | 0 | 0 | 0 | 0 |
| Q271P | 1 | 0 | 0 | 0 | 0 | 0 | 0 | 0 |
| Q271P | 0 | 0 | 0 | 0 | 0 | 0 | 0 | 0 |
| Q271P | 0 | 0 | 0 | 0 | 0 | 0 | 0 | 0 |
| R274Q | 0 | 0 | 0 | 0 | 0 | 0 | 0 | 0 |
| R274W | 0 | 0 | 0 | 0 | 0 | 0 | 0 | 0 |
| R274W | 0 | 0 | 0 | 0 | 0 | 0 | 0 | 0 |
| R274W | 0 | 0 | 0 | 0 | 0 | 0 | 0 | 0 |
| R274W | 0 | 0 | 0 | 0 | 0 | 0 | 0 | 0 |
| R274Q | 0 | 0 | 0 | 0 | 0 | 0 | 0 | 0 |
| R274Q | 0 | 0 | 0 | 0 | 0 | 0 | 1 (4) | HLH post visceral leishmaniasis |
| R274Q | 0 | 0 | 0 | 0 | 0 | 0 | 0 | 0 |
| R274Q | 1 | 1 | 1 | 0 | 0 | 1 | 0 | 0 |
| R274W | 0 | 0 | 0 | 0 | 0 | 0 | 0 | 0 |
| R274Q | 0 | 0 | 0 | 0 | 0 | 0 | 0 | 0 |
| R274Q | 0 | 0 | 0 | 0 | 0 | 0 | 0 | 0 |
| R274Q | 0 | 0 | 0 | 0 | 0 | 0 | 0 | 0 |
| R274Q | 0 | 0 | 0 | 0 | 0 | 0 | 0 | 0 |
| R274Q | 0 | 0 | 0 | 0 | 0 | 0 | 0 | 0 |
| R274Q | 0 | 0 | 0 | 0 | 0 | 0 | 0 | 0 |
| R274Q | 0 | 0 | 0 | 0 | 0 | 0 | 0 | 0 |
| R274W | 1 | 0 | 0 | 0 | 0 | 0 | 0 | 0 |
| R274Q | 0 | 0 | 0 | 0 | 0 | 0 | 0 | 0 |
| R274W | 0 | 1 | 0 | 0 | 0 | 0 | 0 | 0 |
| R274Q | 0 | 1 | 0 | 0 | 0 | 0 | 0 | 0 |
| R274W | 1 | 0 | 0 | 0 | 0 | 0 | 0 | 0 |
| R274W | 0 | 0 | 0 | 0 | 0 | 0 | 0 | 0 |
| R274Q | 0 | 0 | 0 | 0 | 0 | 0 | 0 | 0 |
| R274W | 0 | 1 | 0 | 0 | 0 | 1 | 1 (50) | Accidental |
| R274Q | 0 | 0 | 0 | 0 | 0 | 0 | 0 | 0 |
| R274Q | 1 | 1 | 0 | 0 | 0 | 0 | 0 | 0 |
| R274Q | 0 | 0 | 0 | 1 | 0 | 1 | 0 | 0 |
| R274Q | 0 | 0 | 0 | 0 | 0 | 0 | 0 | 0 |
| R274Q | 0 | 0 | 0 | 0 | 0 | 0 | 0 | 0 |
| R274Q | 0 | 0 | 0 | 0 | 0 | 0 | 0 | 0 |
| R274Q | 0 | 0 | 0 | 0 | 0 | 0 | 0 | 0 |
| R274W | 0 | 0 | 0 | 0 | 0 | 0 | 0 | 0 |
| R274W | 0 | 1 | 1 | 1 | 0 | 0 | 1 (25) | Intracranial haemorrhage |
| R274W | 0 | 0 | 0 | 0 | 0 | 0 | 0 | 0 |
| R274Q | 0 | 1 | 1 | 0 | 0 | 0 | 0 | 0 |
| R274Q | 0 | 0 | 0 | 0 | 0 | 0 | 0 | 0 |
| R274W | 0 | 0 | 0 | 0 | 1 | 1 | 1 (53) | Carcinoma |
| R274W | 0 | 0 | 0 | 0 | 0 | 0 | 0 | 0 |
| R274Q | 0 | 1 | 0 | 0 | 0 | 1 | 0 | 0 |
| R274Q | 0 | 0 | 0 | 0 | 0 | 0 | 0 | 0 |
| R274Q | 1 | 0 | 0 | 0 | 0 | 0 | 0 | 0 |
| R274Q | 0 | 0 | 0 | 0 | 0 | 1 | 0 | 0 |
| R274W | 0 | 0 | 0 | 0 | 0 | 0 | 0 | 0 |
| R274W | 0 | 0 | 0 | 0 | 0 | 0 | 0 | 0 |
| R274Q | 0 | 0 | 0 | 0 | 0 | 0 | 0 | 0 |
| R274Q | 0 | 0 | 0 | 0 | 0 | 0 | 0 | 0 |
| R274Q | 0 | 1 | 0 | 0 | 0 | 0 | 0 | 0 |
| R274Q | 0 | 1 | 0 | 0 | 0 | 0 | 0 | 0 |
| R274Q | 0 | 1 | 0 | 0 | 0 | 0 | 0 | 0 |
| R274Q | 0 | 1 | 0 | 0 | 0 | 0 | 0 | 0 |
| R274Q | 0 | 0 | 0 | 0 | 0 | 0 | 0 | 0 |
| R274W | 0 | 0 | 0 | 0 | 0 | 1 | 0 | 0 |
| R274W | 0 | 0 | 0 | 0 | 0 | 1 | 0 | 0 |
| R274Q | 0 | 0 | 0 | 0 | 0 | 0 | 0 | 0 |
| R274W | 0 | 1 | 1 | 0 | 0 | 0 | 0 | 0 |
| R274Q | 0 | 0 | 0 | 0 | 0 | 0 | 0 | 0 |
| R274Q | 0 | 0 | 0 | 0 | 0 | 0 | 0 | 0 |
| R274Q | 1 | 0 | 0 | 0 | 0 | 0 | 0 | 0 |
| R274Q | 0 | 0 | 0 | 0 | 0 | 0 | 0 | 0 |
| R274Q | 0 | 0 | 0 | 0 | 0 | 0 | 0 | 0 |
| R274Q | 0 | 0 | 0 | 0 | 0 | 0 | 0 | 0 |
| R274Q | 0 | 0 | 1 | 1 | 0 | 1 | 0 | 0 |
| R274Q | 0 | 0 | 0 | 0 | 0 | 0 | 0 | 0 |
| R274Q | 0 | 0 | 1 | 1 | 0 | 0 | 0 | 0 |
| R274Q | 0 | 1 | 0 | 0 | 1 | 1 | 0 | 0 |
| R274W | 0 | 1 | 0 | 0 | 0 | 0 | 0 | 0 |
| R274W | 1 | 1 | 1 | 0 | 0 | 0 | 0 | 0 |
| R274W | 1 | 0 | 0 | 0 | 0 | 0 | 0 | 0 |
| R274W | 0 | 0 | 0 | 0 | 0 | 0 | 0 | 0 |
| R274W | 0 | 0 | 0 | 0 | 0 | 0 | 0 | 0 |
| R274G | 0 | 0 | 0 | 0 | 0 | 0 | 0 | 0 |
| R274W | 0 | 0 | 0 | 0 | 0 | 0 | 0 | 0 |
| R274W | 0 | 0 | 0 | 0 | 0 | 1 | 0 | 0 |
| R274W | 1 | 0 | 0 | 1 | 0 | 0 | 1 (34) | Intracranial haemorrhage |
| R274Q | 1 | 1 | 0 | 0 | 0 | 0 | 0 | 0 |
| R274W | 0 | 0 | 0 | 0 | 0 | 0 | 0 | 0 |
| R274Q | 0 | 0 | 0 | 0 | 0 | 0 | 0 | 0 |
| R274Q | 0 | 0 | 0 | 0 | 0 | 0 | 0 | 0 |
| R274W | 0 | 0 | 0 | 0 | 0 | 0 | 0 | 0 |
| R274W | 1 | 1 | 0 | 0 | 0 | 0 | 0 | 0 |
| R274W | 1 | 1 | 0 | 0 | 0 | 0 | 0 | 0 |
| R274W | 1 | 0 | 0 | 0 | 0 | 0 | 0 | 0 |
| R274W | 0 | 0 | 1 | 0 | 0 | 0 | 0 | 0 |
| R274W | 0 | 1 | 0 | 0 | 0 | 0 | 0 | 0 |
| R274W | 0 | 0 | 0 | 0 | 1 | 0 | 1 (43) | Carcinoma |
| R274W | 1 | 1 | 1 | 0 | 0 | 0 | 0 | 0 |
| R274W | 1 | 0 | 1 | 0 | 0 | 1 | 0 | 0 |
| R274W | 1 | 0 | 0 | 0 | 0 | 0 | 0 | 0 |
| R274W | 0 | 0 | 0 | 0 | 0 | 0 | 0 | 0 |
| R274W | 0 | 0 | 0 | 0 | 0 | 0 | 0 | 0 |
| R274Q | 0 | 0 | 0 | 0 | 0 | 0 | 0 | 0 |
| R274W | 0 | 0 | 0 | 0 | 0 | 0 | 0 | 0 |
| R274W | 0 | 0 | 0 | 0 | 0 | 0 | 0 | 0 |
| R274W | 0 | 0 | 0 | 0 | 0 | 0 | 0 | 0 |
| R274Q | 0 | 0 | 0 | 0 | 0 | 0 | 0 | 0 |
| R274Q | 1 | 0 | 1 | 0 | 0 | 0 | 0 | 0 |
| R274Q | 0 | 0 | 0 | 0 | 0 | 0 | 0 | 0 |
| R274W | 0 | 1 | 0 | 0 | 1 | 0 | 0 | 0 |
| R274W | 0 | 0 | 0 | 0 | 0 | 0 | 0 | 0 |
| R274Q | 0 | 1 | 0 | 0 | 0 | 0 | 0 | 0 |
| K278E | 0 | 0 | 0 | 0 | 0 | 0 | 0 | 0 |
| L280W | 0 | 0 | 0 | 0 | 0 | 0 | 0 | 0 |
| L283F | 0 | 1 | 0 | 0 | 0 | 1 | 0 | 0 |
| L283V | 0 | 0 | 0 | 0 | 0 | 0 | 0 | 0 |
| L283S | 1 | 0 | 0 | 0 | 0 | 1 | 0 | 0 |
| L283M | 1 | 0 | 0 | 0 | 0 | 0 | 0 | 0 |
| L283M | 0 | 0 | 0 | 0 | 0 | 1 | 0 | 0 |
| E284K | 0 | 0 | 0 | 0 | 0 | 0 | 0 | 0 |
| E284K | 0 | 1 | 1 | 0 | 0 | 0 | 0 | 0 |
| E284K | 0 | 0 | 0 | 0 | 0 | 0 | 0 | 0 |
| Q285R | 1 | 1 | 0 | 0 | 0 | 1 | 0 | 0 |
| Q285R | 1 | 0 | 0 | 0 | 0 | 0 | 0 | 0 |
| Q285K | 0 | 0 | 0 | 0 | 0 | 0 | 0 | 0 |
| Q285K | 0 | 0 | 0 | 0 | 0 | 0 | 0 | 0 |
| K286I | 0 | 0 | 0 | 1 | 0 | 0 | 1 (7) | intracranial haemorrhage |
| K286I | 0 | 0 | 0 | 0 | 0 | 1 | 0 | 0 |
| K286I | 0 | 0 | 0 | 0 | 1 | 1 | 1 (52) | Infection |
| Y287D | 0 | 0 | 0 | 0 | 0 | 0 | 0 | 0 |
| Y287N | 0 | 0 | 0 | 0 | 0 | 0 | 0 | 0 |
| Y287H | 0 | 0 | 0 | 0 | 0 | 0 | 0 | 0 |
| Y287D | 1 | 0 | 0 | 0 | 0 | 1 | 0 | 0 |
| T288I | 0 | 0 | 0 | 0 | 0 | 0 | 0 | 0 |
| T288A | 1 | 1 | 0 | 0 | 0 | 1 | 0 | 0 |
| T288N | 0 | 0 | 0 | 1 | 0 | 0 | 0 | 0 |
| T288P | 0 | 0 | 0 | 0 | 0 | 0 | 0 | 0 |
| T288A | 0 | 1 | 0 | 0 | 0 | 1 | 0 | 0 |
| T288A | 0 | 0 | 1 | 0 | 0 | 0 | 0 | 0 |
| T288A | 0 | 0 | 0 | 0 | 0 | 0 | 0 | 0 |
| T288A | 0 | 0 | 0 | 0 | 0 | 0 | 0 | 0 |
| T288A | 0 | 0 | 0 | 0 | 0 | 0 | 0 | 0 |
| Y289H | 1 | 0 | 1 | 0 | 0 | 1 | 0 | 0 |
| Y289C | 0 | 1 | 0 | 0 | 0 | 0 | 0 | 0 |
| Y289C | 0 | 1 | 0 | 0 | 0 | 0 | 0 | 0 |
| Y289C | 0 | 0 | 1 | 0 | 0 | 0 | 0 | 0 |
| Y289C | 0 | 0 | 0 | 1 | 0 | 0 | 0 | 0 |
| Y289C | 0 | 0 | 0 | 0 | 0 | 0 | 0 | 0 |
| Y289C | 0 | 0 | 0 | 0 | 0 | 0 | 0 | 0 |
| Y289C | 0 | 0 | 0 | 0 | 0 | 0 | 0 | 0 |
| Y289C | 0 | 0 | 0 | 0 | 0 | 0 | 0 | 0 |
| Y289C | 0 | 0 | 0 | 0 | 0 | 0 | 0 | 0 |
| Y289C | 0 | 0 | 0 | 0 | 0 | 0 | 0 | 0 |
| Y289C | 0 | 0 | 0 | 0 | 0 | 0 | 0 | 0 |
| D292N | 0 | 0 | 0 | 0 | 0 | 0 | 0 | 0 |
| D292E | 0 | 1 | 0 | 0 | 0 | 1 | 1 (10) | Post-HSCT |
| P293L | 0 | 0 | 0 | 0 | 0 | 1 | 0 | 0 |
| P293L | 0 | 1 | 1 | 0 | 0 | 0 | 0 | 0 |
| P293S | 0 | 0 | 0 | 0 | 0 | 0 | 0 | 0 |
| P293S | 1 | 0 | 0 | 0 | 0 | 0 | 0 | 0 |
| P293S | 0 | 0 | 0 | 0 | 0 | 1 | 0 | 0 |
| P293T | 0 | 0 | 0 | 0 | 0 | 1 | 0 | 0 |
| I294T | 1 | 1 | 0 | 0 | 0 | 1 | 1 (17) | Infection - JC encephalitis |
| I294T | 0 | 1 | 0 | 0 | 0 | 1 | 0 | 0 |
| I294T | 1 | 1 | 0 | 0 | 0 | 1 | 0 | 0 |
| T295K | 0 | 0 | 0 | 0 | 0 | 1 | 0 | 0 |
| N297del | 0 | 0 | 0 | 0 | 0 | 1 | 0 | 0 |
| K298N | 0 | 0 | 0 | 0 | 0 | 0 | 0 | 0 |
| K298N | 0 | 0 | 0 | 0 | 0 | 0 | 0 | 0 |
| K298N | 0 | 0 | 0 | 0 | 0 | 0 | 0 | 0 |
| L301del | 1 | 1 | 0 | 0 | 0 | 0 | 0 | 0 |
| R321S | 0 | 0 | 1 | 0 | 0 | 1 | 0 | 0 |
| R321S | 0 | 0 | 0 | 0 | 0 | 1 | 1 (55) | Cardiac arrest |
| R321G | 0 | 0 | 0 | 0 | 0 | 1 | 0 | 0 |
| R321G | 0 | 0 | 0 | 0 | 0 | 0 | 0 | 0 |
| R321G | 0 | 0 | 0 | 0 | 0 | 0 | 0 | 0 |
| R321S | 0 | 0 | 0 | 0 | 0 | 0 | 0 | 0 |
| R321S | 1 | 1 | 0 | 0 | 0 | 0 | 0 | 0 |
| R321S | 1 | 1 | 0 | 0 | 0 | 0 | 0 | 0 |
| R321S | 0 | 1 | 0 | 0 | 0 | 0 | 0 | 0 |
| R321S | 1 | 1 | 0 | 0 | 0 | 0 | 0 | 0 |
| R321S | 0 | 1 | 0 | 0 | 0 | 1 | 1 (22) | Hepatitis |
| R321S | 1 | 1 | 0 | 0 | 0 | 0 | 0 | 0 |
| R321S | 0 | 1 | 0 | 0 | 0 | 0 | 0 | 0 |
| C324R | 0 | 0 | 0 | 0 | 0 | 0 | 0 | 0 |
| C324R | 0 | 0 | 0 | 0 | 0 | 0 | 0 | 0 |
| C324R | 1 | 0 | 0 | 0 | 0 | 0 | 0 | 0 |
| C324R | 0 | 0 | 0 | 0 | 0 | 0 | 0 | 0 |
| C324R | 1 | 1 | 0 | 0 | 0 | 0 | 0 | 0 |
| C324R | 0 | 0 | 0 | 0 | 0 | 0 | 0 | 0 |
| C324R | 0 | 0 | 0 | 0 | 0 | 0 | 0 | 0 |
| C324R | 1 | 1 | 0 | 0 | 0 | 0 | 1 (25) | Alveolar haemorrhage |
| C324F | 0 | 0 | 0 | 0 | 0 | 0 | 1 (3) | Post-HSCT |
| C324Y | 0 | 1 | 0 | 0 | 0 | 0 | 0 | 0 |
| M325K | 1 | 0 | 0 | 0 | 0 | 1 | 1 (15) | Infection |
| H328R | 1 | 1 | 0 | 0 | 0 | 0 | 0 | 0 |
| H328R | 1 | 1 | 0 | 0 | 0 | 1 | 0 | 0 |
| H328R | 1 | 1 | 0 | 0 | 0 | 1 | 0 | 0 |
| P329L | 1 | 0 | 0 | 0 | 0 | 0 | 0 | 0 |
| P329L | 0 | 1 | 0 | 0 | 0 | 0 | 0 | 0 |
| P329R | 0 | 0 | 0 | 0 | 0 | 1 | 0 | 0 |
| P329L | 0 | 0 | 0 | 0 | 0 | 0 | 0 | 0 |
| P329L | 0 | 0 | 0 | 0 | 0 | 0 | 0 | 0 |
| Q330K | 1 | 1 | 0 | 0 | 0 | 1 | 0 | 0 |
| G338V | 0 | 0 | 0 | 0 | 0 | 0 | 0 | 0 |
| G338V | 0 | 0 | 1 | 0 | 0 | 0 | 0 | 0 |
| Q340P | 0 | 0 | 0 | 0 | 0 | 0 | 0 | 0 |
| K344E | 0 | 0 | 1 | 0 | 0 | 1 | 0 | 0 |
| K344E | 1 | 0 | 0 | 0 | 0 | 0 | 1 (5) | Respiratory failure due to COVID-19 infection |
| K344E | 0 | 0 | 0 | 0 | 0 | 1 | 0 | 0 |
| K344Q | 0 | 0 | 1 | 0 | 0 | 1 | 0 | 0 |
| L351F | 1 | 1 | 0 | 0 | 0 | 1 | 0 | 0 |
| L351F | 0 | 0 | 0 | 0 | 0 | 1 | 0 | 0 |
| L351F | 1 | 0 | 0 | 0 | 0 | 0 | 0 | 0 |
| L351F | 1 | 1 | 0 | 0 | 0 | 1 | 0 | 0 |
| L351F | 0 | 0 | 0 | 1 | 0 | 1 | 0 | 0 |
| L351F | 1 | 1 | 0 | 0 | 0 | 1 | 0 | 0 |
| L351F | 1 | 0 | 0 | 0 | 0 | 0 | 0 | 0 |
| E353K | 0 | 0 | 0 | 0 | 0 | 0 | 0 | 0 |
| E353K | 0 | 0 | 0 | 1 | 0 | 1 | 0 | 0 |
| E353K | 0 | 0 | 0 | 0 | 0 | 0 | 1 (22) | Unknown |
| E353K | 0 | 0 | 0 | 0 | 0 | 0 | 0 | 0 |
| E353K | 0 | 0 | 0 | 0 | 0 | 0 | 0 | 0 |
| E353K | 0 | 0 | 0 | 0 | 0 | 0 | 0 | 0 |
| L354M | 1 | 0 | 0 | 0 | 0 | 0 | 0 | 0 |
| L354M | 1 | 0 | 0 | 0 | 0 | 1 | 0 | 0 |
| L354V | 0 | 1 | 0 | 0 | 0 | 0 | 0 | 0 |
| N355D | 0 | 0 | 0 | 0 | 0 | 1 | 0 | 0 |
| N355D | 0 | 0 | 1 | 0 | 0 | 0 | 0 | 0 |
| N355D | 0 | 0 | 0 | 1 | 0 | 1 | 0 | 0 |
| N357D | 0 | 0 | 0 | 0 | 0 | 0 | 0 | 0 |
| N357D | 0 | 0 | 0 | 0 | 0 | 0 | 0 | 0 |
| N357D | 0 | 0 | 0 | 0 | 0 | 0 | 0 | 0 |
| N357D | 0 | 0 | 0 | 0 | 0 | 0 | 0 | 0 |
| N357D | 0 | 0 | 0 | 0 | 0 | 0 | 0 | 0 |
| N357D | 0 | 0 | 0 | 0 | 0 | 0 | 0 | 0 |
| L358F | 0 | 0 | 0 | 1 | 0 | 0 | 1 (16) | Pulmonary haemorrhage |
| L358F | 0 | 1 | 0 | 0 | 0 | 1 | 0 | 0 |
| L358W | 1 | 1 | 0 | 1 | 0 | 1 | 1 (4) | Subarachnoid haemorrhage |
| L358W | 1 | 1 | 0 | 1 | 0 | 1 | 1 (4) | Intracranial haemorrhage |
| E370D | 0 | 0 | 0 | 0 | 0 | 0 | 0 | 0 |
| G384C | 0 | 0 | 0 | 0 | 0 | 0 | 1 (1) | Infection |
| G384D | 1 | 1 | 0 | 0 | 0 | 1 | 0 | 0 |
| G384D | 0 | 0 | 0 | 0 | 0 | 0 | 0 | 0 |
| G384D | 0 | 0 | 1 | 0 | 0 | 1 | 0 | 0 |
| G384D | 0 | 0 | 0 | 0 | 0 | 0 | 0 | 0 |
| G384D | 0 | 0 | 0 | 0 | 0 | 0 | 0 | 0 |
| T385M | 0 | 0 | 0 | 0 | 0 | 0 | 0 | 0 |
| T385M | 0 | 1 | 0 | 0 | 0 | 1 | 1 (15) | Infection |
| T385M | 1 | 1 | 1 | 0 | 0 | 1 | 0 | 0 |
| T385M | 1 | 0 | 0 | 0 | 0 | 0 | 0 | 0 |
| T385M | 1 | 1 | 0 | 0 | 0 | 1 | 1 (10) | Post-HSCT |
| T385M | 1 | 1 | 0 | 0 | 0 | 1 | 1 (20) | Infection |
| T385M | 1 | 0 | 0 | 0 | 0 | 1 | 1 (32) | Infection |
| T385M | 1 | 1 | 0 | 0 | 0 | 1 | 1 (7) | Infection |
| T385M | 0 | 0 | 0 | 0 | 0 | 0 | 0 | 0 |
| T385M | 0 | 0 | 0 | 0 | 0 | 0 | 1 (2) | Infection (pneumonia) |
| T385M | 0 | 0 | 0 | 0 | 0 | 1 | 0 | 0 |
| T385M | 1 | 0 | 0 | 0 | 0 | 1 | 0 | 0 |
| T385M | 0 | 0 | 0 | 0 | 0 | 0 | 0 | 0 |
| T385M | 0 | 0 | 0 | 0 | 0 | 1 | 0 | 0 |
| T385M | 0 | 0 | 0 | 0 | 0 | 1 | 0 | 0 |
| T385M | 0 | 0 | 1 | 0 | 0 | 1 | 0 | 0 |
| T385M | 0 | 0 | 0 | 0 | 0 | 0 | 1 (9) | Infection |
| T385M | 0 | 1 | 1 | 0 | 0 | 1 | 0 | 0 |
| T385M | 0 | 0 | 0 | 0 | 0 | 1 | 0 | 0 |
| T385M | 0 | 0 | 0 | 0 | 0 | 0 | 1 (1) | Infection |
| T385M | 1 | 1 | 0 | 0 | 0 | 1 | 0 | 0 |
| T385M | 0 | 0 | 0 | 0 | 0 | 0 | 0 | 0 |
| T385M | 0 | 1 | 0 | 0 | 0 | 1 | 0 | 0 |
| T385M | 0 | 1 | 0 | 0 | 0 | 0 | 0 | 0 |
| T385M | 0 | 1 | 0 | 0 | 0 | 1 | 1 (19) | Cerebral haemorrhage |
| T385M | 0 | 0 | 0 | 0 | 0 | 0 | 0 | 0 |
| T385M | 0 | 0 | 1 | 0 | 0 | 1 | 0 | 0 |
| T385M | 0 | 0 | 0 | 0 | 0 | 0 | 0 | 0 |
| T385M | 1 | 0 | 0 | 0 | 0 | 0 | 0 | 0 |
| T385M | 1 | 0 | 0 | 0 | 0 | 1 | 0 | 0 |
| T385M | 0 | 0 | 0 | 0 | 0 | 0 | 0 | 0 |
| T385M | 0 | 1 | 0 | 0 | 0 | 0 | 0 | 0 |
| T385M | 0 | 1 | 0 | 0 | 0 | 0 | 0 | 0 |
| T385M | 0 | 0 | 0 | 0 | 0 | 0 | 0 | 0 |
| T385M | 0 | 1 | 0 | 0 | 0 | 0 | 0 | 0 |
| T385M | 0 | 0 | 0 | 0 | 0 | 1 | 0 | 0 |
| T385M | 0 | 0 | 0 | 0 | 0 | 0 | 0 | 0 |
| T385M | 1 | 0 | 1 | 0 | 0 | 1 | 0 | 0 |
| T385M | 0 | 0 | 1 | 0 | 0 | 1 | 0 | 0 |
| T385M | 0 | 0 | 0 | 0 | 0 | 1 | 0 | 0 |
| T385M | 0 | 0 | 0 | 0 | 0 | 1 | 0 | 0 |
| T385M | 0 | 1 | 1 | 1 | 0 | 1 | 1 (16) | Infection |
| T385M | 1 | 0 | 0 | 0 | 0 | 1 | 0 | 0 |
| T385M | 1 | 1 | 0 | 0 | 0 | 1 | 0 | 0 |
| T385M | 0 | 1 | 0 | 1 | 0 | 1 | 1 (19) | Haemorrhage |
| T385M | 0 | 1 | 0 | 0 | 0 | 0 | 0 | 0 |
| T385M | 0 | 1 | 0 | 0 | 0 | 0 | 1 (20) | Infection |
| T385M | 0 | 0 | 0 | 0 | 0 | 1 | 0 | 0 |
| T385M | 0 | 0 | 0 | 0 | 0 | 0 | 0 | 0 |
| T385M | 0 | 0 | 0 | 0 | 0 | 1 | 0 | 0 |
| T385M | 0 | 1 | 0 | 0 | 0 | 1 | 0 | 0 |
| T385K | 0 | 0 | 0 | 0 | 0 | 0 | 0 | 0 |
| T385M | 0 | 1 | 0 | 1 | 0 | 1 | 1 (19) | Disseminated CMV, Pancytopaenia with coagulopathy, terminal CNS bleed |
| T387A | 0 | 0 | 1 | 0 | 0 | 0 | 0 | 0 |
| T387A | 0 | 0 | 0 | 0 | 0 | 0 | 0 | 0 |
| T387A | 0 | 0 | 0 | 0 | 0 | 0 | 0 | 0 |
| T387A | 0 | 0 | 0 | 0 | 0 | 0 | 0 | 0 |
| K388E | 1 | 0 | 0 | 0 | 0 | 0 | 0 | 0 |
| K388E | 1 | 0 | 1 | 0 | 0 | 0 | 0 | 0 |
| K388E | 0 | 0 | 0 | 0 | 0 | 0 | 0 | 0 |
| K388E | 0 | 0 | 0 | 0 | 0 | 0 | 0 | 0 |
| K388E | 0 | 0 | 1 | 0 | 1 | 0 | 0 | 0 |
| K388E | 1 | 1 | 0 | 0 | 1 | 0 | 0 | 0 |
| K388E | 0 | 0 | 0 | 0 | 0 | 0 | 0 | 0 |
| K388E | 0 | 0 | 0 | 0 | 0 | 0 | 0 | 0 |
| K388E | 0 | 0 | 1 | 0 | 0 | 0 | 0 | 0 |
| K388E | 1 | 0 | 1 | 0 | 0 | 0 | 0 | 0 |
| K388E | 0 | 0 | 0 | 0 | 0 | 0 | 0 | 0 |
| K388E | 0 | 0 | 0 | 0 | 0 | 0 | 0 | 0 |
| K388E | 1 | 0 | 0 | 0 | 0 | 0 | 0 | 0 |
| K388E | 1 | 0 | 0 | 0 | 0 | 0 | 1 (40) | Infection |
| K388E | 0 | 0 | 0 | 0 | 0 | 0 | 0 | 0 |
| V389A | 0 | 0 | 0 | 0 | 0 | 0 | 1 (10) | Accidental |
| V389L | 0 | 0 | 0 | 0 | 0 | 0 | 0 | 0 |
| M390T | 0 | 0 | 0 | 0 | 0 | 0 | 0 | 0 |
| M390I | 0 | 0 | 0 | 0 | 0 | 0 | 0 | 0 |
| M390I | 0 | 0 | 0 | 0 | 0 | 0 | 0 | 0 |
| M390T | 0 | 0 | 0 | 0 | 0 | 1 | 0 | 0 |
| M390T | 0 | 0 | 0 | 0 | 0 | 0 | 0 | 0 |
| M390I | 0 | 0 | 0 | 0 | 0 | 0 | 1 (4) | Infection |
| M390T | 1 | 0 | 0 | 0 | 0 | 0 | 0 | 0 |
| M390T | 1 | 0 | 0 | 0 | 0 | 1 | 1 (30) | Post-HSCT |
| M392T | 0 | 0 | 0 | 0 | 0 | 0 | 0 | 0 |
| M392T | 0 | 0 | 0 | 0 | 0 | 0 | 0 | 0 |
| M392T | 1 | 0 | 0 | 0 | 0 | 0 | 0 | 0 |
| M392T | 0 | 1 | 0 | 0 | 0 | 1 | 0 | 0 |
| N397D | 0 | 0 | 0 | 0 | 0 | 0 | 0 | 0 |
| N397D | 0 | 0 | 0 | 0 | 0 | 0 | 1 (2) | Post-HSCT |
| L400Q | 0 | 0 | 1 | 0 | 0 | 0 | 0 | 0 |
| L400V | 0 | 1 | 0 | 1 | 1 | 0 | 0 | 0 |
| L400V | 0 | 0 | 0 | 0 | 0 | 1 | 0 | 0 |
| L400Q | 0 | 0 | 0 | 0 | 0 | 0 | 0 | 0 |
| F404Y | 0 | 0 | 0 | 0 | 0 | 0 | 0 | 0 |
| F404Y | 0 | 0 | 0 | 0 | 0 | 0 | 0 | 0 |
| F404Y | 0 | 1 | 0 | 0 | 0 | 0 | 0 | 0 |
| F404V | 0 | 0 | 0 | 0 | 0 | 1 | 0 | 0 |
| L407V | 0 | 0 | 0 | 0 | 0 | 0 | 0 | 0 |
| L407V | 0 | 0 | 1 | 0 | 0 | 0 | 0 | 0 |
| G416R | 1 | 1 | 0 | 0 | 0 | 1 | 0 | 0 |
| T419R | 0 | 0 | 0 | 0 | 0 | 1 | 0 | 0 |
| T419K | 0 | 0 | 0 | 0 | 0 | 1 | 0 | 0 |
| T419R | 0 | 0 | 0 | 0 | 0 | 0 | 0 | 0 |
| T419R | 0 | 0 | 0 | 0 | 0 | 0 | 0 | 0 |
| T437I | 0 | 0 | 0 | 0 | 0 | 0 | 0 | 0 |
| T437N | 0 | 0 | 0 | 0 | 1 | 0 | 0 | 0 |
| T437N | 0 | 0 | 0 | 0 | 1 | 0 | 0 | 0 |
| S462R | 0 | 0 | 0 | 0 | 0 | 0 | 0 | 0 |
| S462R | 1 | 1 | 1 | 0 | 0 | 0 | 0 | 0 |
| S462R | 0 | 0 | 0 | 0 | 0 | 0 | 0 | 0 |
| S462R | 0 | 0 | 0 | 0 | 0 | 0 | 0 | 0 |
| S466R | 0 | 1 | 0 | 1 | 0 | 1 | 0 | 0 |
| S466R | 0 | 1 | 0 | 0 | 0 | 1 | 0 | 0 |
| S466R | 0 | 0 | 1 | 0 | 0 | 1 | 0 | 0 |
| S466R | 1 | 0 | 0 | 0 | 0 | 0 | 0 | 0 |
| W468R | 1 | 0 | 0 | 0 | 0 | 0 | 0 | 0 |
| D517G | 0 | 0 | 0 | 0 | 0 | 0 | 0 | 0 |
| C543R | 0 | 1 | 0 | 0 | 0 | 0 | 0 | 0 |
| C543R | 0 | 1 | 0 | 0 | 0 | 0 | 0 | 0 |
| E545K | 0 | 1 | 0 | 0 | 0 | 1 | 0 | 0 |
| E559G | 1 | 0 | 0 | 0 | 0 | 0 | 0 | 0 |
| E563Q | 1 | 0 | 1 | 0 | 0 | 1 | 1 (4) | Multiorgan failure in the setting of HLH secondary to COVID-19 infection |
| N574I | 0 | 1 | 1 | 0 | 0 | 1 | 0 | 0 |
| N574H | 0 | 0 | 0 | 0 | 0 | 1 | 0 | 0 |
| E609K | 0 | 0 | 0 | 0 | 0 | 0 | 1 (10) |  |
| E609K | 0 | 1 | 0 | 0 | 0 | 1 | 0 | 0 |
| H629Y | 0 | 0 | 0 | 0 | 0 | 1 | 0 | 0 |
| H629Y | 1 | 1 | 0 | 0 | 0 | 0 | 0 | 0 |
| V653I | 1 | 1 |  | 0 | 0 | 0 | 0 | 0 |
| V653I | 1 | 0 | 0 | 0 | 0 | 0 | 0 | 0 |
| N658S | 0 | 0 | 0 | 0 | 1 | 1 | 0 | 0 |
| E705Q | 0 | 1 | 0 | 0 | 0 | 0 | 0 | 0 |
| E705V | 1 | 1 | 0 | 0 | 1 | 0 | 1 (36) | SCC |
| S708F | 0 | 1 | 0 | 0 | 0 | 1 | 0 | 0 |
| E711Q | 0 | 0 | 0 | 0 | 0 | 0 | 0 | 0 |
| E711Q | 0 | 0 | 0 | 0 | 0 | 0 | 0 | 0 |
| E711Q | 1 | 1 | 0 | 0 | 0 | 0 | 0 | 0 |
| E711Q | 0 | 1 | 0 | 0 | 0 | 0 | 0 | 0 |
| E711Q | 0 | 0 | 0 | 0 | 0 | 0 | 0 | 0 |
| E711Q | 0 | 0 | 0 | 0 | 0 | 0 | 0 | 0 |
| E711Q | 0 | 1 | 0 | 0 | 0 | 1 | 0 | 0 |
| E711Q | 0 | 0 | 0 | 0 | 1 | 0 | 1 (85) | Carcinoma |
| T720I | 0 | 1 | 0 | 1 | 0 | 0 | 0 | 0 |
| T720I | 0 | 0 | 0 | 0 | 1 | 0 | 0 | 0 |
| T720I | 0 | 1 | 0 | 0 | 1 | 0 | 0 | 0 |
| T720I | 0 | 1 | 0 | 1 | 0 | 0 | 0 | 0 |
| P725L | 0 | 0 | 0 | 0 | 0 | 0 | 0 | 0 |
| P725L | 0 | 0 | 1 | 0 | 0 | 0 | 0 | 0 |
| P725L | 0 | 0 | 0 | 0 | 0 | 0 | 0 | 0 |
| P725L | 0 | 0 | 0 | 0 | 0 | 1 | 0 | 0 |
